# Reversible Nucleolar Complex Coacervation by Short Cationic Peptides

**DOI:** 10.1021/jacs.6c06046

**Published:** 2026-06-24

**Authors:** Maximilian Schuler, Emirhan Koca, Leon Driehaus-Ortiz, Marius G. Braun, Albin Lahu, Anna-Lena Holtmannspötter, Ha-Chi Nguyen, Job Boekhoven, David Y. W. Ng, Tanja Weil

**Affiliations:** † Max Planck Institute for Polymer Research, Ackermannweg 10, D-55128 Mainz, Germany; ‡ Max Planck School Matter to Life, Jahnstraße 29, D-69120 Heidelberg, Germany; § Department of Bioscience, School of Natural Sciences, 9184 Technical University of Munich, Lichtenbergstrasse 4, D-85748 Garching, Germany

## Abstract

Biomolecular condensates formed through liquid–liquid phase separation play central roles in intracellular organization and regulation. Replicating such dynamic compartmentalization using minimal synthetic components remains exceptionally challenging inside living cells. Here, we report short cationic peptides that undergo directed complex coacervation in living cells through preferential interactions with endogenous polyanionic biomolecules. Although the peptides were designed to contain a mitochondrial targeting motif, the Arg residues and cellular RNA guide the supramolecular interactions toward selective enrichment of liquid-like coacervates in nucleolar regions. In vitro studies reveal that polymeric RNA mimics promote coacervation far more efficiently than ATP, establishing RNA–peptide interactions as the principal driving force. In cells, nucleolar complex coacervates form rapidly and exhibit liquid-like behavior with fast molecular exchange. Importantly, the assemblies are transient and reversible: sustained peptide supply maintains the condensed state, whereas substrate depletion triggers droplet dissolution and recovery of cellular function. These findings demonstrate that endogenous biopolymer distributions can guide and participate in the formation of synthetic coacervates with minimalistic peptides, achieving reversible reorganization of intracellular components. More broadly, this work provides a framework for engineering synthetic coacervates with nonequilibrium, life-like features that operate in direct exchange with living cellular environments.

## Graphical abstract

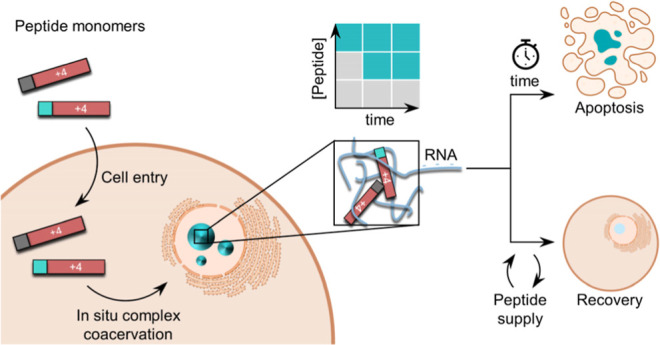

## Introduction

Biomolecular condensates formed through liquid–liquid phase separation organize intracellular chemistry without membranes, enabling dynamic control over molecular localization, reaction rates, and cellular signaling.^1^ These membrane-less compartments emerge in defined subcellular regions, including stress granules in the cytoplasm and nucleoli in the nucleus, where they regulate processes ranging from stress response to RNA metabolism to ribosome biogenesis.^2,3^ Inspired by these dynamic cellular assemblies, substantial efforts have focused on engineering synthetic condensates inside living cells.^4–15^ However, achieving intracellular phase separation using minimal synthetic building blocks that engage and integrate endogenous cellular components remains a major challenge.

At the material–biology interface, intracellular self-assembly (ISA) promoted by rationally designed self-assembling peptides has emerged as a powerful method for generating nanostructures inside living cells.^16–23^ Following cellular entry in their monomeric form, endogenous triggers activate monomer precursors and initiate their assembly into polymers.^24^ The diverse intracellular microenvironments act as stimulus reservoirs (including pH, ROS, glutathione, and enzymes), spatially controlling the respective supramolecular chemistry within a specific location to form aggregates, nanofibers, and hydrogels, with applications ranging from programmed cellular death to stimulation of cytotoxic T cells.^25,26^ Despite these advances, extending the ISA strategy from liquid–solid to liquid–liquid phase transitions remains largely unexplored due to significant conceptual and practical challenges. Reaching the critical concentration for liquid–liquid phase separation (LLPS) is particularly demanding, as it necessitates local peptide monomer concentrations that often exceed those required for designed liquid-to-solid phase transitions by at least 1 order of magnitude.^27–34^ Moreover, the dynamic nature of these droplets renders them inherently delicate, as environmentally sensitive interactions can readily be disrupted by variations in salt concentration, pH, or temperature.^35^ Consequently, the design of complex coacervates demands high-affinity interactions, a constant flux of the scaffold material, and robust stability, requirements that are especially critical in the cellular context, where environmental properties fluctuate in both time and space.^36,37^ Owing to these practical limitations, peptide-based coacervates have so far been employed primarily in their preformed state, either as drug delivery vehicles or as artificial catalytic compartments.^7,38–40^ Recent advances in engineered biomolecular condensates in living cells have enabled programmable sequestration, intracellular control, and de novo condensate design, yet these systems have largely relied on proteins or preorganized scaffolds rather than in situ complex coacervation of short synthetic peptides with endogenous cellular components.^8,41,42^

However, without forming these coacervates in situ, the dynamic and adaptive behavior characteristic of natural condensates cannot be directly accessed or interrogated within the full complexity of the cellular environment.^43^ Initiated by RNA–protein interactions, P granules, for instance, undergo multiple assembly and disassembly cycles during cell division, which is indispensable for symmetry-breaking events in *C. elegans* model systems.^44^ Inspired by this conceptual complexity, the substrate-induced complex coacervation strategy introduced here offers new opportunities to actively interact with cellular states and metabolism. By establishing dynamic intracellular reservoirs that assemble and dissipate in response to substrate availability, such systems could enable reversible modulation of intracellular function. In this context, developing a synergistic framework that integrates synthetic and natural matter under nonequilibrium conditions provides a missing link between systems chemistry and cell-responsive biomaterials.^20^

Here, we demonstrate that short cationic peptides can exploit endogenous RNA-rich environments to form nucleolar complex coacervates in living cells, revealing a targeting principle governed by intracellular biopolymer distributions rather than solely by installed organelle-targeting motifs. These peptides serve as structural substrates that form complex coacervates in situ within living cells within 1 min. By correlating in vitro and in vivo data, we identify RNA affinity and the peptide’s Arg-content in the cellular context as key determinants of subcellular localization. Notably, under substrate-rich conditions, cells transiently maintain a dynamic regime in which droplet persistence reflects the continuous interplay between assembly and dissipation. Upon prolonged exposure, however, cells fail to sustain this state due to mitochondrial dysfunction and cell membrane permeabilization at the onset of apoptosis. In contrast, under substrate-depleted conditions, the deactivation pathway mediated by cellular expulsion of the substrate is sufficient to promote compound clearance and enable cellular recovery. This reversibility contrasts sharply with thermodynamically controlled, aggregate-based systems and highlights the nonequilibrium, life-like nature of the installed complex coacervates. Collectively, these findings establish ISA as a novel design principle for the nonequilibrium formation of coacervates at the interface of materials and biology, with both therapeutic and fundamental implications.

## Results and Discussion

### Peptide Design, Synthesis, and Purification

We designed a short cationic peptide Fmoc-K­(TPP)­RGRGR-CONH_2_ (**Fmoc**-**R** ^**TPP**^) with TPP (triphenyl phosphonium) introduced to promote mitochondrial targeting (Figure 1).^45^ Arg residues were selected because their guanidino groups provide positive charges under physiological conditions, thereby enabling charge–charge and cation–π interactions with multivalent counterions such as ATP or nucleic acids.^46^ Three Arg residues were employed as previous studies in pristine systems have demonstrated their strong tendency to form complex coacervates in the presence of suitable counterions.^47^ Alternating Arg with Gly residues mimics the RG motifs commonly observed in phase-separating RNA-binding proteins.^46–48^ Ultimately, the N-terminal 9-fluorenylmethoxycarbonyl (Fmoc) group restricts rotational freedom, engages in cation–π interactions, and serves as a hydrophobic π-block that stabilizes droplets under high salt concentration.^49–51^ To facilitate fluorescence imaging, the Coumarin-343 derivative Coumarin-K­(TPP)­RGRGR-CONH_2_ (**Coum**-**R**_**3**_^**TPP**^) was additionally employed (Figure 1). The peptides were synthesized by standard Fmoc-based solid-phase-peptide-synthesis (SPPS). TPP was coupled to the resin by selective side-chain deprotection of the labile 4-methyltrityl protecting group on Lys ε-NH_2_ (Scheme S1).^52^ All peptides were purified by reversed-phase high-performance liquid chromatography (HPLC), and their purity was confirmed by liquid chromatography mass spectrometry (LCMS) (Figure S1a–d).

**Figure 1:**
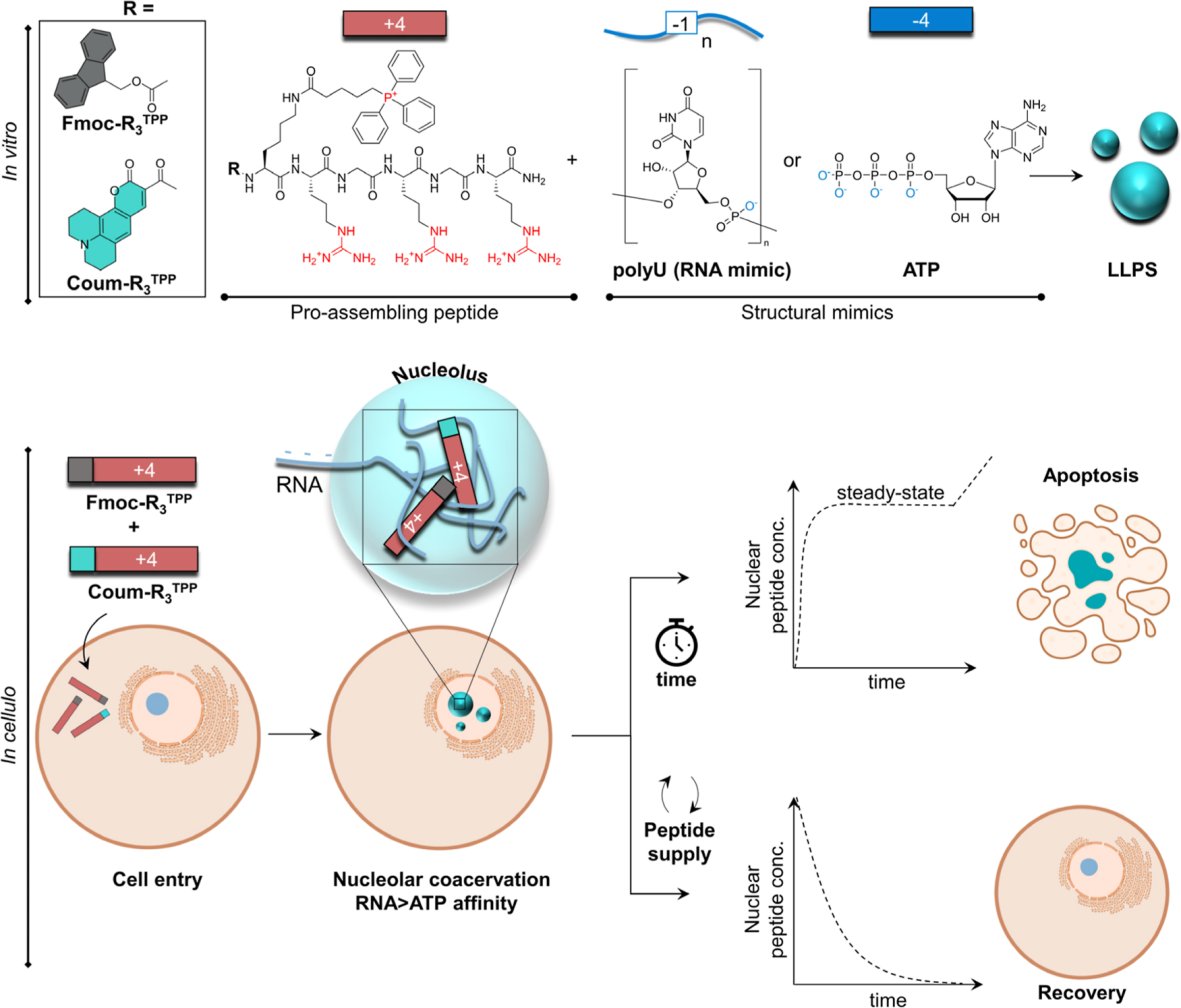
Conceptual framework for intracellular complex coacervation induced by short cationic peptides. In vitro, cationic pro-assembling peptides bearing different N-terminal modifications undergo liquid–liquid phase separation through charge–charge interactions with negatively charged cellular mimics such as polyU (RNA mimic) or ATP. In cellulo, soluble peptide monomers first enter the cell and subsequently accumulate in the nucleolus, where complex coacervation occurs through interactions with endogenous RNA. Under peptide-abundant conditions, nucleolar droplets persist dynamically before prolonged exposure leads to metabolic dysfunction and apoptotic cell death. In contrast, upon peptide depletion (e.g., medium exchange), complex coacervates dissolve and cellular morphology and viability recover. This schematic illustrates how intracellular peptide influx and removal regulate reversible phase separation in living cells.

### LLPS through Complex Coacervation In Vitro

We anticipated that the addition of **Fmoc**-**R**_**3**_^**TPP**^ to either ATP or polyuridylic acid (polyU) would lead to complex coacervation in vitro. Indeed, mixing **Fmoc**- **R**_**3**_^**TPP**^ with either of these counterions produced a macroscopic increase in turbidity under neutral aqueous conditions (Dulbecco’s phosphate-buffered saline (DPBS) buffer, pH 7.4) (Figures 2a,b and S2). The presence of both constituents (**Fmoc**-**R**_**3**_^**TPP**^ and either ATP or polyU) was required to induce turbidity, thereby excluding homocoacervation (Figure S3).

**Figure 2:**
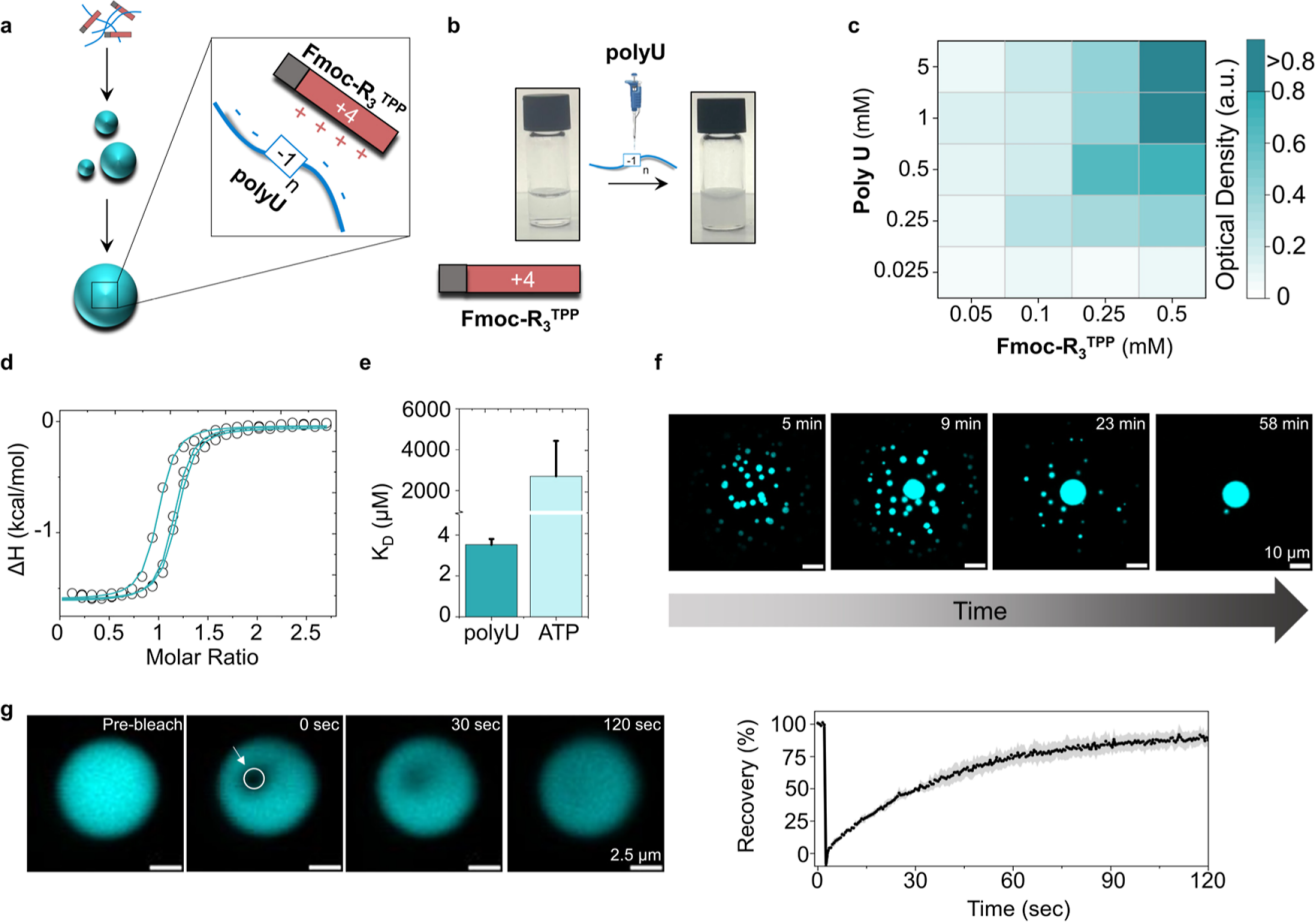
In vitro complex coacervation of **Fmoc**-**R**_**3**_^**TPP**^ with multivalent counterions. (a) Schematic illustration of complex coacervation between the cationic peptide **Fmoc**-**R**_**3**_^**TPP**^ and polyanionic counterions. (b) Macroscopic turbidity observed upon mixing **Fmoc**-**R**_**3**_^**TPP**^ with polyU; [**Fmoc**-**R**_**3**_^**TPP**^] = 250 μM, [polyU] = 1 mM. (c) Turbidity phase diagram showing droplet formation as a function of peptide and polyU concentration (optical density at 600 nm). As representative data points, mean values of *n* = 3 replicas are presented. (d) Isothermal titration calorimetry (ITC) profiles of **Fmoc**-**R**_**3**_^**TPP**^ interacting with polyU. Molar ratios are normalized to charges. Three curves correspond to *n* = 3 replicas. (e) Dissociation constants (K_D_) derived from ITC measurements showing substantially stronger binding to polyU compared to ATP. Error bars represent mean ± s.d. from *n* = 3 replicas. (f) Time-dependent CLSM images showing time-dependent coalescence of droplets formed by **Fmoc**-**R**_**3**_^**TPP**^ (230 μΜ), **Coum**-**R**_**3**_^**TPP**^ (20 μM), and polyU (1 mM). (g) Left: fluorescence recovery after photobleaching (FRAP) demonstrating liquid-like dynamics of the droplets. Right: normalized fluorescence recovery curve (mean ± s.d. from *n* = 3 replicas) for **Fmoc**-**R**_**3**_^**TPP**^ (230 μM), **Coum**-**R**_**3**_^**TPP**^ (20 μM), polyU (1 mM). All experiments were conducted in DPBS buffer, pH 7.4.

Expectedly, polyU exhibited a stronger tendency toward droplet formation than ATP as a polyanion. Whereas at least 1.5 mM **Fmoc**-**R**_**3**_^**TPP**^ was required to induce droplet formation in the presence of 4 mM ATP, only 0.05 mM peptide was sufficient in the presence of 250 μM polyU (Figures 2c and S4). To probe the relative interaction strengths of ATP + **Fmoc**-**R**_**3**_^**TPP**^ and polyU + **Fmoc**-**R**_**3**_^**TPP**^, the stability of the resulting droplets upon dilution with DPBS or acetonitrile was assessed. While droplets formed by polyU + **Fmoc**-**R**_**3**_^**TPP**^ resisted dilution (up to 1:2 v/v), those formed by ATP + **Fmoc**-**R**_**3**_^**TPP**^ readily dissolved (Figure S5a,b). A fluorescent centrifugation-based pull-down assay was performed to quantify the partitioning of **Coum**-**R**_**3**_^**TPP**^ into droplets formed with polyU or ATP in the presence of **Fmoc**-**R**_**3**_^**TPP**^. Supplementation of **Fmoc**-**R**_**3**_^**TPP**^ with 8 vol % of **Coum**-**R**_**3**_^**TPP**^ did not measurably affect its coacervation behavior (Figure S5c). Because droplets formed by **Fmoc**-**R**_**3**_^**TPP**^ and ATP were insufficiently stable to be pelleted upon centrifugation (16.2k g), **Fmoc**-**R**_**3**_^**TPP**^ was approximately 47-fold more likely to partition into droplets formed by polyU (Figure S5d).

Isothermal titration calorimetry (ITC) further substantiated these findings when **Fmoc**-**R**_**3**_^**TPP**^ was titrated against polyU or ATP (Figures 2d and S6a). While **Fmoc**-**R**_**3**_^**TPP**^ showed only weak interactions with ATP, the dissociation constant (*K*_D_) was more than 2 orders of magnitude lower for polyU (*K*_D_ = 3.5 ± 0.3 μM) (Figure 2e). The reaction enthalpy (Δ*H*) was approximately 9-fold higher for polyU (Figure S6b). Moreover, the data revealed a close to equimolar stoichiometry (calculated per charge) for polyU (*N* = 1.1 ± 0.1), which could not be reliably determined for ATP due to a weak signal (Figure S6c). Together, these data identify polyU as the superior counterion in terms of both affinity and stability, which together promote complex coacervation. This observation is consistent with previous reports in which RNA, as a competitive binder, effectively displaced ATP because of its higher charge valency.^53^

To study the dynamics in complex coacervates formed by **Fmoc**-**R**_**3**_^**TPP**^ and polyU, confocal laser scanning microscopy (CLSM) was performed using an in-house-built microreactor as described previously.^54^ Complex coacervates formed instantaneously, followed by coalescence over time (Figure 2f, Movie S1). DLS corroborated these observations, showing a continuous increase in particle size over time (Figure S7). Fluorescence recovery after photobleaching (FRAP) confirmed their liquid-like behavior, with recovery reaching approximately up to ∼90% within 2 min, consistent with previously reported peptide-based systems (Figure 2g).^55,56^ Their dynamic properties were further supported by partitioning experiments, in which sulforhodamine B and chymotrypsin-FITC (CT-FITC) readily partitioned into the liquid droplets (Figure S8). Finally, NaCl, urea, and 1,6-hexanediol screens identified charge–charge interactions and hydrogen bonding rather than hydrophobic interactions as key driving forces for complex coacervation (Figure S9). Collectively, these results establish charge–charge interactions, hydrogen bonding, and multivalent polymeric counterions as the principal determinants of stable complex coacervation in this system.

### Concentration-, Time-, and Gly-Arg-Dependent Intracellular LLPS

After examining potential interaction partners in vitro, we next traced cellular peptide uptake and intracellular droplet formation (Figure 3a). A549 human lung adenocarcinoma cells were incubated with varying amounts of **Fmoc**-**R**_**3**_^**TPP**^ + **Coum**-**R**_**3**_^**TPP**^ (total peptide concentrations: 125 μM, 250 μM, and 500 μM) using a coincubation approach for visualization.^16^ In this design, only the concentration of **Fmoc**-**R**_**3**_^**TPP**^ was varied, while the concentration of **Coum**-**R**_**3**_^**TPP**^ was maintained at a constant concentration of 20 μM. The coassembly was chosen as droplets formed by RNA and **Coum**-**R**_**3**_^**TPP**^ self-quenched due to high fluorophore concentrations inside the complex coacervates, potentially interfering with accurate data interpretation of complex coacervates formed by **Coum**-**R**_**3**_^**TPP**^ alone (Figure S10).^57^ Intracellular coumarin fluorescence appeared in a dose-and time-dependent manner. While negligible signals were detected at 125 μM, cells treated with 250 μM or 500 μM of **Fmoc**-**R**_**3**_^**TPP**^/**Coum**-**R**_**3**_^**TPP**^ displayed detectable fluorescence after approximately 30 and 1 min, respectively (Figure 3b). Interestingly, despite the presence of the typical mitochondria-targeting moiety TPP, fluorescence accumulation occurred predominantly in the nuclear and perinuclear regions rather than in mitochondria, demonstrating an intracellular transport override by the Arg-rich peptide (Figure 3c).^17^ This observation is in agreement with a previous study that described the insufficient mitochondrial targeting properties of single TPP-containing peptides and studies that observed the nuclear targeting capabilities of Arg-rich sequences.^17,58^ Furthermore, this localization is consistent with the stronger affinity of **Fmoc-R_3_^TPP^** for RNA compared to ATP observed in vitro, which favors coacervation in RNA-rich nuclear environments (Figures 2d,e and S5).

**Figure 3:**
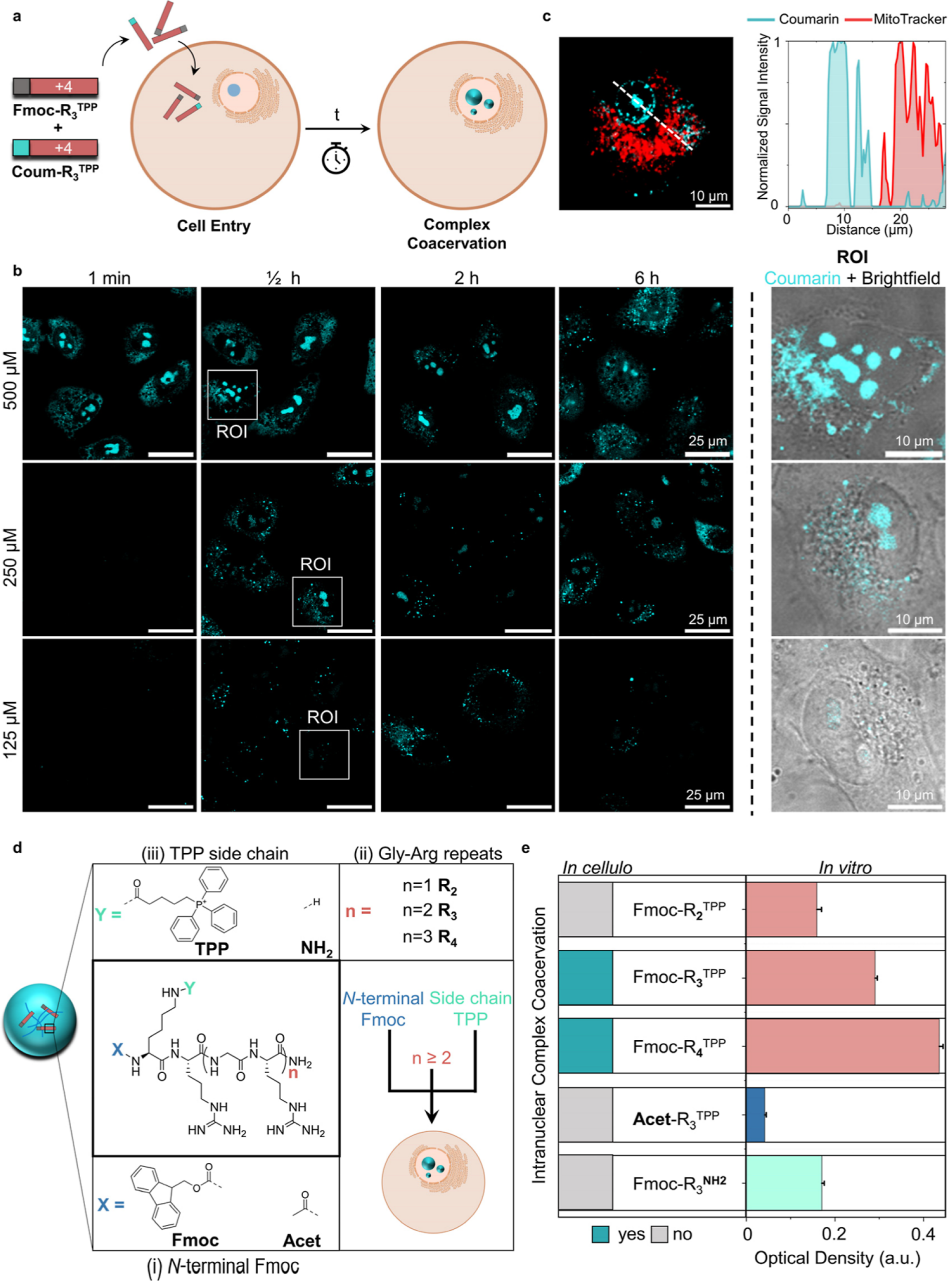
Peptide-driven intracellular complex coacervation. (a) Co-incubated **Fmoc**-**R**_**3**_^**TPP**^/**Coum**-**R**_**3**_^**TPP**^ soluble monomers enter A549 cells and form complex coacervates in RNA-rich nuclear regions, consistent with nucleolar accumulation. (b) Time-dependent CLSM studies with varying **Fmoc**-**R**_**3**_^**TPP**^ concentrations (480 μM, 230 μM, 105 μM) while maintaining **Coum**-**R**_**3**_^**TPP**^ at a constant concentration of 20 μM. Cells were fixed prior to imaging. Coumarin fluorescence is shown in cyan, and the corresponding regions of interest (ROIs) are displayed as bright-field overlays on the right. (c) CLSM analysis of living cells. MitoTracker (red) costaining experiment (left) and the corresponding normalized fluorescence intensity profile along the dashed line (right). Total peptide concentration: 250 μM after 2 h of incubation. Experiments were performed in FBS-free DMEM. (d) Structure–function relationship through variations in Gly–Arg repeats and pendant π-block groups. (e) In cellulo intracellular complex coacervation propensity (left) and in vitro optical density (at 600 nm) of 0.1 mM **Acet**-**R**_**3**_^**TPP**^, **Fmoc**-**R**_**2**_^**TPP**^, **Fmoc**-**R**_**3**_^**TPP**^, **Fmoc**-**R**_**4**_^**TPP**^, and **Fmoc**-**R**_**3**_^**NH2**^ in the presence of 0.25 mM polyU. Standard deviations represent error bars of *n* = 3 replicas.

Having established a preliminary understanding of the forces driving nucleolar complex coacervation, several **Fmoc**-**R**_**3**_^**TPP**^ derivatives were introduced to establish a structure–function relationship. Specifically, these derivatives were designed to elucidate the impact of (i) the N-terminal Fmoc moiety, (ii) the number of Gly–Arg repeats, and (iii) the TPP side chain on droplet formation propensity in vitro and in cellulo (Figures 3d and S1). To assess (i) the role of the N-terminal Fmoc, the acetylated version Ac–K­(TPP)­RGRGR-CONH_2_ (**Acet**-**R**_**3**_^**TPP**^) was introduced. Replacing the Fmoc by its acetylated version shifted the phase diagram in the presence of polyU significantly toward higher peptide concentrations, indicating that the π-character is crucial for complex coacervation (Figures 3e and S11a). This becomes particularly evident in the cellular context, where complex coacervation was completely absent even when incubated at 500 μΜ **Acet**-**R**_**3**_^**TPP**^/**Coum**-**R**_**3**_^**TPP**^ for 6 h (Figures 3e and S12). Next, Fmoc-K­(TPP)­RGR-CONH_2_ = **Fmoc**-**R**_**2**_^**TPP**^ and Fmoc-K­(TPP)­RGRGRGR-CONH_2_ = **Fmoc**-**R**_**4**_^**TPP**^ were synthesized to examine (ii) the impact of the number of Gly–Arg repeats (Figures 3d and S1). In vitro, the polyU-dependent phase diagram revealed a strong correlation between droplet formation propensity and number of Gly–Arg repeats, particularly in the low peptide/RNA concentration regime (Figures 2c and S11b,c). In cellulo, this correlation remained consistent, as nucleolar complex coacervation was observed only for **Fmoc**-**R**_**3**_^**TPP**^ and **Fmoc**-**R**_**4**_^**TPP**^, while **Fmoc**-**R**_**2**_^**TPP**^ samples showed no evidence of coacervation (Figures 3e and S12)_._ As a control experiment, to exclude simple nuclear partitioning of the peptide material as the origin of the fluorescence signal, the coassembling peptide 20 μM **Coum**-**R**_**3**_^**TPP**^ was investigated, which alone showed no localized signal enrichment (Figure S13). Lastly, (iii) the role of the TPP side chain was investigated by synthesizing the TPP-lacking derivative Fmoc-K­(NH_2_)­RGRGR-CONH_2_ = **Fmoc**-**R**_**3**_^**NH2**^ (Figure S1). As similarly observed for the acetylated version **Acet**-**R**_**3**_^**TPP**^, omitting the π-block on the side chain would shift the phase diagram toward higher peptide concentrations (Figures 2c and S11d). This is also reflected in cellulo, where no significant signals were detected after 6 h of incubation at 500 μΜ **Fmoc**-**R**_**3**_^**NH2**^/**Coum**-**R**_**3**_^**TPP**^ (Figures 3e and S12).

These data collectively demonstrate that intranuclear complex coacervation requires (i) an N-terminal Fmoc group, (ii) at least two Gly–Arg repeats, and (iii) the TPP side chain, owing to effective nucleolar peptide concentration governed by the number of Arg residues and droplet formation propensity determined by both π-character and charge (Figure 3d,e). These trends align well with a previous in-cell NMR study that points out the stickiness of poly-Arg peptides in *E. coli* as potential drivers for macromolecular assembly.^59^

### Subcellular Localization, Distribution, and Cellular Response

Having established the correlation between the in vitro droplet-forming propensity and intranuclear LLPS, we proceeded to further elucidate the subcellular localization and impact on cellular function (Figure 4a). Inside the nucleus, the nucleolus constitutes the largest and most prominent membrane-less organelle, where RNA accumulates as a result of ribosomal biogenesis, rRNA transcription, and processing.^3^ This property enriches nucleic acids into densely packed granules inside the nucleolar microenvironment.^60^ Co-staining with the RNA-specific dye SYTO showed that **Fmoc**-**R**_**3**_^**TPP**^/**Coum**-**R**_**3**_^**TPP**^ accumulates in cellular regions enriched in RNA, consistent with nucleolar localization as similarly observed for Arg-rich peptide sequences (Figure 4b).^18^ As the complex coacervates form within the nucleolus, their shapes do not appear perfectly spherical (Figure 4b). Moreover, turbidity measurements using cell lysate showed no significant increase in optical density upon exposure to **Fmoc**-**R**_**3**_^**TPP**^, indicating that (i) nonspecific binding to cytosolic nucleic acids is negligible and (ii) the induction of complex coacervation is reserved for RNA-dense conditions, which are governed by the effective local concentrations of both the peptide and counterions (Figure S14).

**Figure 4:**
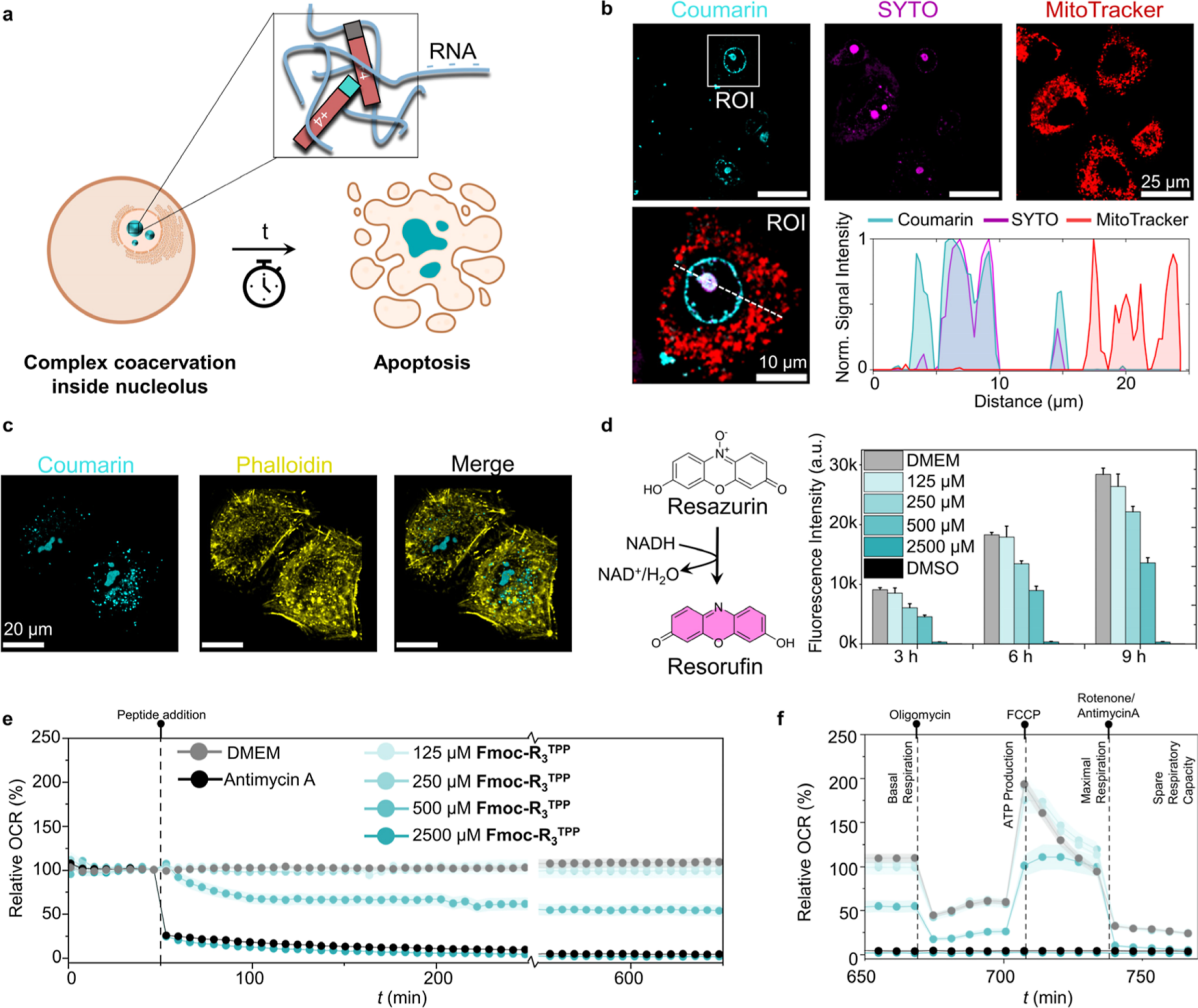
Subcellular localization and cellular response to intranuclear complex coacervation. (a) Monomers of **Fmoc**-**R**_**3**_^**TPP**^/**Fmoc**-**R**_**3**_^**TPP**^ readily enter viable A549 cells, infiltrating the nucleolus to drive complex coacervation in the presence of densely packed RNA. As time proceeds, the in situ LLPS prompts apoptosis. (b) CLSM live-cell costaining experiments with Coumarin (cyan), SYTO (magenta), and MitoTracker (red). Normalized fluorescence is quantified along the dotted line. [**Fmoc**-**R**_**3**_^**TPP**^] = 230 μM, [**Coum**-**R**_**3**_^**TPP**^] = 20 μM after 2 h of incubation. (c) 3D representation of phalloidin (yellow) and Coumarin (cyan) of fixed and costained cells. [**Fmoc**-**R**_**3**_^**TPP**^] = 230 μM, [**Coum**-**R**_**3**_^**TPP**^] = 20 μM after 2 h of incubation. (d) Time-dependent resazurin-based metabolic assay with varying [**Fmoc**-**R**_**3**_^**TPP**^], DMEM-treated negative control, and DMSO-treated positive control. Error bars represent standard deviations from *n* = 6 replicas. (e) Oxygen consumption rate (OCR) of A549 cells treated with varying concentrations of **Fmoc**-**R**_**3**_^**TPP**^. Prior to peptide addition, the OCR is set as 100% as a reference. Error bars represent standard errors of the mean (SEM) from at least *n* = 3 replicas. (f) Mito stress assay of **Fmoc**-**R**_**3**_^**TPP**^-treated A549 cells (labels are in accordance with (e) with electron transport chain modulators. After ATP synthase inhibition (oligomycin), FCCP disrupts the mitochondrial membrane potential. Rotenone and antimycin A then inhibit complexes I and III of the electron transport chain. Error bars represent standard errors of the mean (SEM) from at least *n* = 3 replicas. All experiments were conducted in FBS-free DMEM.

To elucidate the uptake mechanism, peptide-treated cells were incubated at 4 °C. Notably, this condition did not efficiently inhibit peptide uptake as evidenced by complex coacervation at 500 μM **Fmoc**-**R**_**3**_^**TPP**^/**Coum**-**R**_**3**_^**TPP**^ after 2 h of incubation (Figure S15). This data in combination with the observed almost instantaneous complex coacervation hints toward an ATP-independent uptake mechanism as described by other Arg-rich systems.^61–63^ Next, the impact of intranuclear complex coacervation on cellular metabolic rate and viability was assessed. Co-staining experiments with Phalloidin, Propidium Iodide (PI), and MitoTracker revealed preserved cellular morphology and viability-associated features at the emergence of complex coacervation, as indicated by an intact actin cytoskeleton, mitochondria, and cell shape (Figures 4b,c and S16). However, as time proceeded, enhanced cell membrane permeability, cell rounding, and the presence of cell debris suggested progressive loss of viability upon prolonged exposure (Figures 3b and S17a–c). Moreover, the initially localized coumarin signal in the nucleolus gradually distributes throughout the whole cell, which is not only accompanied by nuclear fragmentation but also consistent with membrane permeabilization and loss of compartmental integrity during cell death (Figures 3b and S17d,e).

We performed a resazurin-based metabolic activity assay with varying concentrations of **Fmoc**-**R**_**3**_^**TPP**^ to assess cellular metabolic activity in peptide-treated cells in bulk (Figure 4d). Whereas the metabolic activity at 125 μM was similar to that of DMEM-treated negative controls, it was critically impaired at peptide concentrations >250 μM (Figures 4d and S18a). Additionally, the metabolic activity of **Fmoc**-**R**_**4**_^**TPP**^-treated cells decreased even further, while cells subjected to the negative control peptides **Acet**-**R**_**3**_^**TPP**^, **Fmoc**-**R**_**2**_^**TPP**^, or **Fmoc**-**R**_**3**_^**NH2**^ were negligibly affected after 6 h of incubation at 500 μM (Figure S18b–d). These data further support that the biological impact is specifically associated with complex coacervation rather than peptide exposure alone.

We additionally conducted extracellular flux analyses of mitochondrial function to measure the oxygen consumption rate (OCR) and gain further insights into cellular respiration. In line with the resazurin assay, the OCR similarly showed **Fmoc**-**R**_**3**_^**TPP**^ concentration dependency (Figure 4e). Whereas cells treated with 125 μM **Fmoc**-**R**_**3**_^**TPP**^ exhibited an OCR of 103.4 ± 8.8% after approximately 11 h, it drastically dropped to 54.1 ± 6.8% and 0.7 ± 0.5%, respectively, when 500 μM or 2500 μM was applied instead (Figure 4e). A mito stress test was performed to elucidate the impact of complex coacervation on ATP production and spare respiratory capacity (Figure 4f). Upon treatment with electron transport chain modulators, samples at 500 μM and 2500 μM showed a decrease in both parameters, suggesting gradual mitochondrial dysfunction leading to cell death (Figure 4f).

Next, we introduced Annexin-V to ascertain the downstream pathway as a consequence of mitochondrial dysfunction. The protein binds to phosphatidylserine located on the external leaflet of the membrane when cells undergo apoptosis. In the presence of Annexin V-FITC, cells displayed clear membrane-associated signals already at the onset of coacervate formation (1 min at 500 μM **Fmoc**-**1** ^**TPP**^) (Figure S19a). Thus, apoptotic signaling appears to be initiated rapidly even while gross cellular morphology remains initially preserved. Prolonging the incubation time to 1 h further enriched PI in the nuclear region, supporting progression from early apoptotic features toward loss of membrane integrity at later stages (Figure S19a). The protein profiler immunoassay revealed expression changes in Bcl-2, Survivin, TNF, HSP 27, TRAIL, and p27 at 1 h, suggesting multiple apoptotic pathways are affected on a systemic level (Figure S19b).

Together, these data link sustained intranuclear complex coacervation to mitochondrial dysfunction, metabolic collapse, and apoptosis-associated cell death.

### Dynamic Persistence and Reversibility of Intranuclear Complex Coacervation

Liquid condensates are highly dynamic assemblies in which constituent molecules (proteins/RNA) continuously and rapidly exchange with the surrounding dilute (bulk) phase.^64^ Moreover, many of these assemblies are coupled to energy fluxes that sustain their liquid state and thereby enable transient signaling and regulation.^44,65^ To probe whether the nucleolar complex coacervates formed by **Fmoc**-**R**_**3**_^**TPP**^ share these dynamic characteristics, we conducted live-cell FRAP experiments. To prevent the continuous influx of peptide monomers, the dynamics were first examined under substrate-depleted conditions in which 1 min treatment with 500 μM **Fmoc**-**R**_**3**_^**TPP**^/**Coum**-**R**_**3**_^**TPP**^ was followed by a medium exchange procedure (Figure 5a). Notably, the fluorescence signal recovered within approximately 6 s after bleaching, supporting the liquid-like behavior (Figure 5b,c). Under substrate-depleted conditions, the overall fluorescence intensity decreased to 78.5 ± 1.5% in the absence of bleach-point correction, indicating that peptide replenishment from the surrounding medium was strongly limited (Figure 5c). Consistent with this interpretation, CLSM live-cell imaging of cells treated with 500 μM **Fmoc**-**R**_**3**_^**TPP**^/**Coum**-**R**_**3**_^**TPP**^ under the same substrate-depleted conditions further corroborated the dynamic behavior of the droplets (Figure 5d,e). Initially, prominent intranuclear droplets and cell blebbing (white arrows) were detected, suggesting that the onset of coacervation-induced stress is accompanied by transient detachment of the actin cortex from the plasma membrane (Figure 5d).^66^

**Figure 5:**
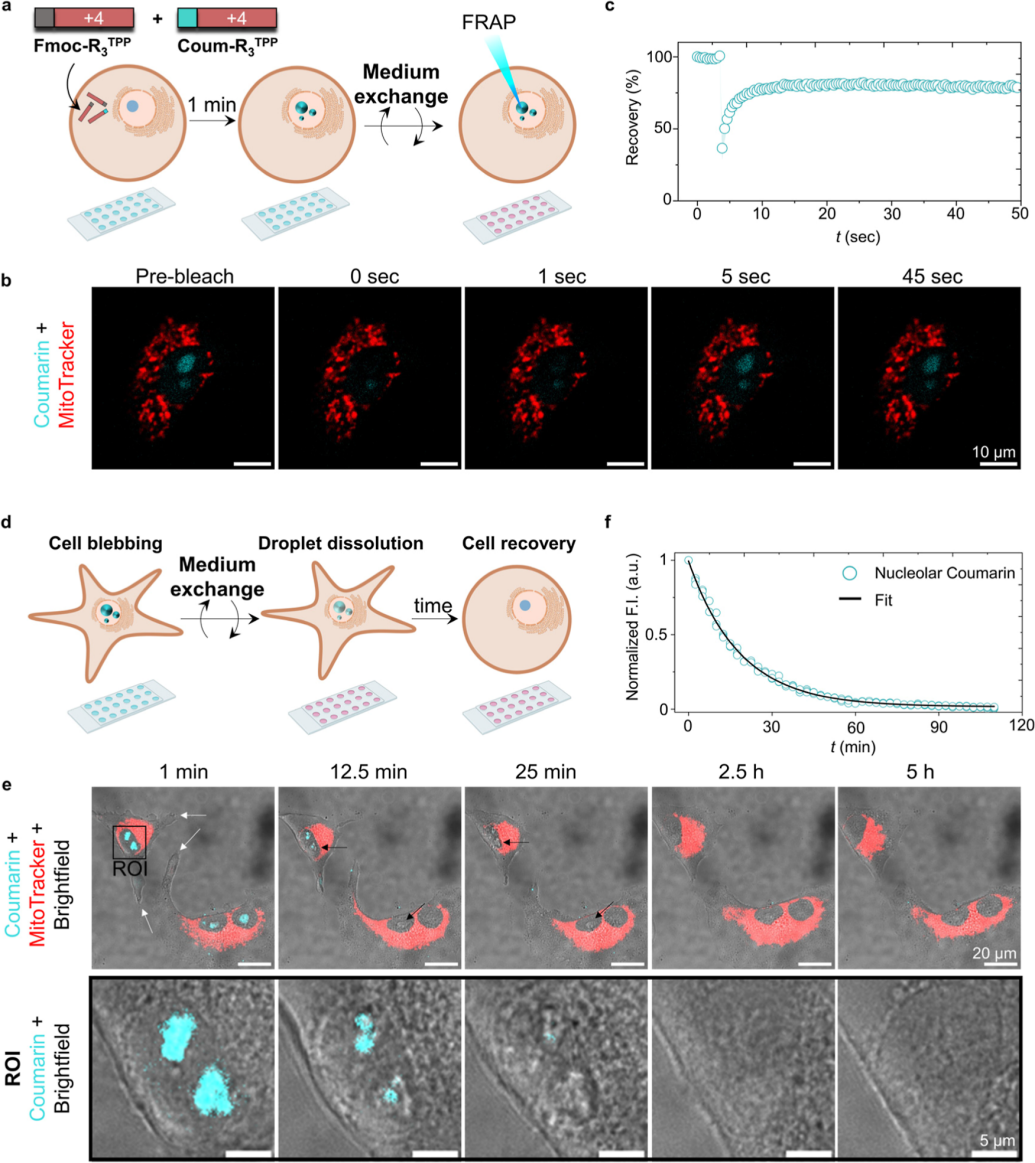
Droplet dynamics and cell recovery under substrate-depleted conditions. (a) In-cell FRAP experiments including a medium exchange step. (b) Representative live-cell images of A549 cells, which undergo the FRAP procedure. [**Fmoc**-**R**_**3**_^**TPP**^] = 480 μM, [**Coum**-**R**_**3**_^**TPP**^] = 20 μM. (c) FRAP recovery curves of peptide-treated A549 cells under substrate-depleted conditions. Error bars (too small to visualize) represent standard deviations from *n* = 6 replicas. (d) Recovery experiments where cells are first treated for 1 min with [**Fmoc**-**R**_**3**_^**TPP**^] = 480 μM, [**Coum**-**R**_**3**_^**TPP**^] = 20 μM, which readily prompts LLPS inside the nucleolus. The stress component triggers cell blebbing, which can be recovered by medium exchange within a time frame of 5 h. (e) Time-dependent CLSM of cells after treatment and media exchange. White and black arrows highlight cell blebbing and a change in nuclear morphology, respectively. (f) Quantification of nucleolar Coumarin signal over time after medium exchange. The average of *n* = 3 droplets from two cells is fitted and depicted as a black line. All experiments were conducted in FBS-free DMEM.

However, the localized intracellular droplet signal diminished over time (at ∼60 min), after which cells gradually recovered a viable morphology within 5 h, as evidenced by changes in refractive index and re-establishment of cellular adhesion and spreading (Figure 5e,f). Moreover, morphological changes of the nuclear region (black arrows) suggested intracellular reorganization following compound treatment and droplet dissolution (Figure 5e). From this point onward, cells that had eliminated the peptide resumed division, indicating the restoration of biological function (Figure S20). These observations were further substantiated by cell viability measurements. Cells treated with 500 μM **Fmoc**-**R**_**3**_^**TPP**^ for 24 h exhibited a viability of 17.0 ± 12.0%. In stark contrast, applying the medium exchange protocol restored cellular viability to 79.6 ± 7.7% after 24 h, further emphasizing that peptide removal under substrate-depleted conditions enables droplet dissolution and cellular recovery (Figure S21).

Intrigued by this reversibility, we next examined the system under substrate-sustained, steady-state conditions (Figure 6a). Using the same FRAP parameters as under substrate depletion (Figure 5a–c), fluorescence recovery increased to 98.6 ± 11.8% after 50 s, indicating continued exchange of the peptide material within the nucleolar complex coacervates when the extracellular peptide remained available (Figure 6a–c). To further assess the extent of this dynamic behavior, the same cell was subjected to multiple rounds of FRAP (Figure 6d). The fluorescence signal recovered over as many as six cycles without showing significant loss of recovery amplitude, showing that complex coacervates can be maintained dynamically as long as peptide supply is sustained (Figures 6d and 5c).

**Figure 6:**
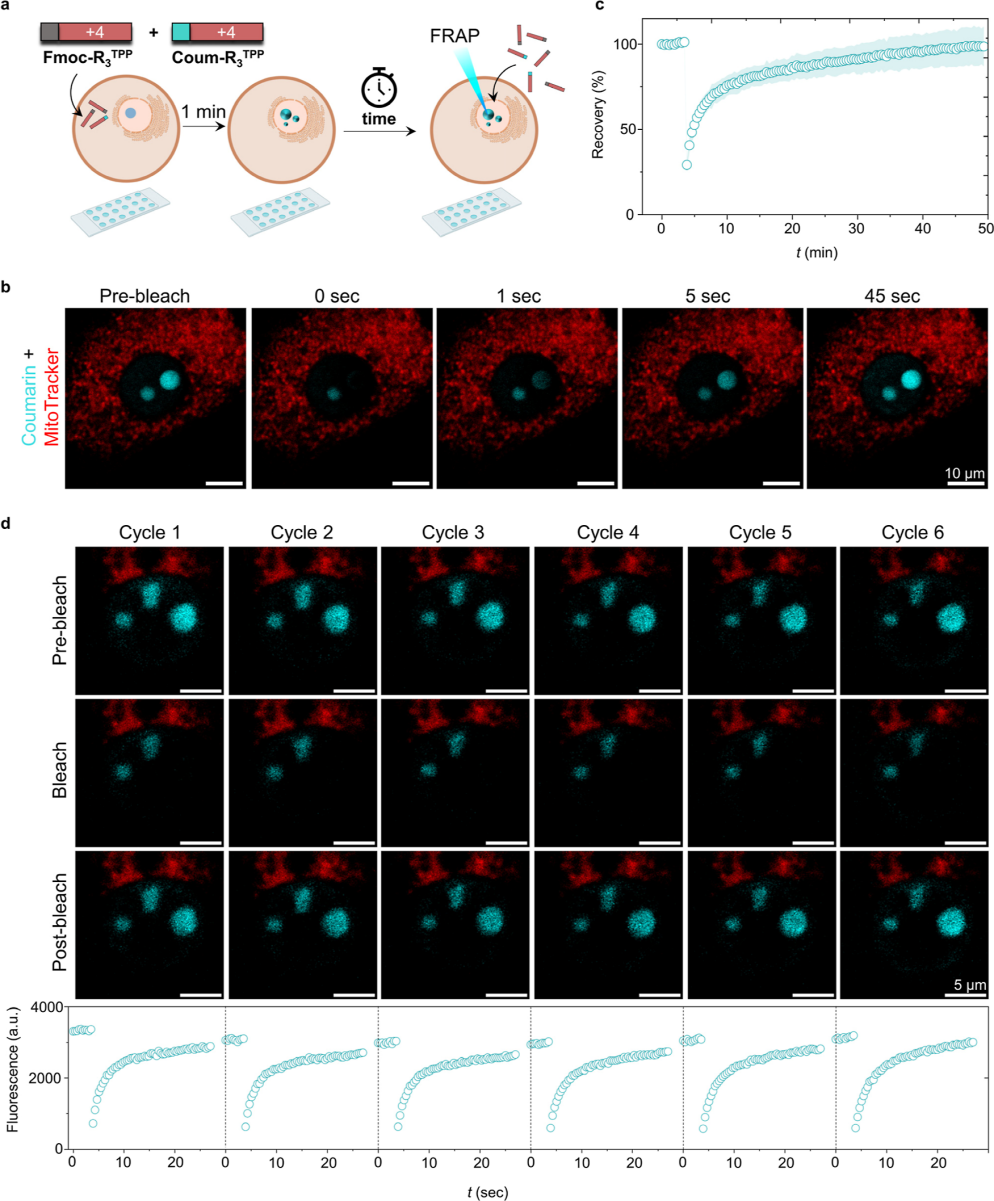
Droplet dynamics under steady-state conditions. (a) In-cell FRAP experiments under substrate-abundant (steady-state) conditions. (b) Representative FRAP live-cell images of A549 cells treated with [**Fmoc**-**R**_**3**_^**TPP**^] = 480 μM, [**Coum**-**R**_**3**_^**TPP**^] = 20 μM. (c) FRAP recovery curves of peptide-treated A549 cells. Error bars represent standard deviations from *n* = 9 replicas (d). Repeated FRAP cycles on a single droplet in peptide-treated A549 cells. The same experimental conditions were applied as in (a). All experiments were conducted in FBS-free DMEM.

Together, these results define a reversible intracellular coacervation window in which nucleolar complex coacervates persist dynamically under continued peptide supply but dissolve upon substrate depletion, thereby allowing for recovery of cellular function.

## Conclusions

We have shown that short cationic peptides can form complex coacervates inside living cells through preferential interactions with endogenous polyanionic biomolecules. Rather than being governed primarily by the installed targeting motif, subcellular localization is redirected by the Arg-rich nature of the peptide and intracellular interaction landscape, leading to selective enrichment of liquid-like complex coacervates in RNA-rich nucleolar regions. In this way, the present system establishes a targeting principle based not only on molecular ligands but also on the spatial distribution of endogenous biopolymers within the cell. Importantly, the resulting assemblies are dynamic, transient, and reversible and therefore more closely resemble native cellular condensates than previously reported intracellular peptide assemblies that culminate in persistent fiber formation or irreversible stress states. By distinguishing substrate-sustained coacervate persistence from substrate-depleted dissolution and recovery, this work further identifies a reversible regime in which intracellular complex coacervates can be installed, maintained temporarily, and subsequently removed. This nonequilibrium steady state is maintained by a constant turnover of peptide monomers, which the cell can withstand only for a limited time before undergoing apoptosis (Figure S22). These findings establish minimal synthetic peptides as context-responsive building blocks for intracellular coacervates and provide a conceptual foundation for future strategies in reversible compartment engineering, transient metabolic modulation, and the creation of synthetic reaction spaces that operate in direct exchange with living cellular environments.

## Supplementary Material





## Notes

Additional description of methods and supplementary data showing LCMS data of all synthesized peptides; control experiments including turbidity assays of **Fmoc**-**R**_**3**_^**TPP**^ with ATP or individual components; supplementary droplet stability assays and ITC data; assays to determine level of unspecific binding and cellular uptake mechanism; phase diagrams, confocal imaging, and cell cytotoxicity assays for control peptides; and cell apoptosis assays and cell recovery studies after peptide removal (PDF)
Time-lapse imaging and coalescence of complex coacervates (MP4)
