# Supplementary material for: Reversible Nucleolar Complex Coacervation by Short Cationic Peptides
Source: J Am Chem Soc. 2026 Jun 24;148(26):27528–39. doi: 10.1021/jacs.6c06046 (PMC13352621; doi:10.1021/jacs.6c06046)
Supplement: Supplementary file 1 [file ja6c06046_si_001.pdf]

Reversible Nucleolar Complex Coacervation by Short Cationic Peptides

Maximilian Schuler[a][b], Emirhan Koca[a][b], Leon Driehaus-Ortiz, [a] Marius G. Braun[a], Albin Lahu[a], Anna-Lena Holtmannspötter[c], Ha-Chi Nguyen[a], Job Boekhoven[b][c], David Y.W. Ng*[a], Tanja Weil*[a][b]

[a] Max Planck Institute for Polymer Research, Ackermannweg 10, D-55128 Mainz, Germany.
[b] Max Planck School Matter to Life, Jahnstraße 29, D-69120 Heidelberg, Germany.
[c] Department of Bioscience, School of Natural Sciences, Technical University of Munich, Lichtenbergstrasse 4, D-85748 Garching, Germany.
Email: david.ng@mpip-mainz.mpg.de, weil@mpip-mainz.mpg.de

1 Materials, Instruments and Methods 4
1.1 Materials 4
1.2. Instruments and Methods 4
1.2.1. Liquid Chromatography-Mass Spectrometry (LC-MS) 4
1.2.2. *In vitro* droplet formation 4
1.2.3.Turbidity measurements 4
1.2.4. Pull-down measurements 4
1.2.5. Isothermal Titration Calorimetry (ITC) 4
1.2.6. Dynamic Light Scattering (DLS) 5
1.2.7. *In vitro* Confocal-laser scanning microscopy (CLSM) 5
1.2.8. Fluorescence Recovery After Photobleaching (FRAP) 5
1.2.9. *In cellulo* CLSM 6
1.2.9.1. Live Cell Imaging 6
1.2.9.2. Co-stains 6
1.2.10. Resazurin-based cell metabolism assay 7
1.2.11. Cell viability assay 7
1.2.12. Extracellular flux experiments 7
1.2.13. Annexin-V staining 8
1.2.14. Apoptosis array 8
1.2.15. Cell lysate binding assay 9
1.3. Synthesis and purification 10
1.3.1. Synthesis of short cationic peptides 10
1.3.2. Purification of peptides 10
2. Supplementary data 12

1 Materials, Instruments and Methods

1.1 Materials

All solvents and reagents were purchased from commercial vendors and if not stated otherwise utilized without further purification. Synthesis grade reagents were employed for the syntheses of peptides. High performance liquid chromatography (HPLC) was conducted using acetonitrile (ACN) as HPLC grade. MilliQ-H_2O was gathered from the Millipore purification system by *Merck Millipore*.

1.2. Instruments and Methods

1.2.1. Liquid Chromatography-Mass Spectrometry (LC-MS)

Compounds were analyzed by HPLC–ESI–MS by a Shimadzu LCMS-2020 system using Kinetex EVO C18 column (2.6 µm, 100 Å, 50 × 2.1 mm). Milli-Q water (0.1% formic acid) and ACN (0.1% formic acid) were applied as the mobile phases. The gradient started at 5% ACN and 95% aqueous phase and was held for 2 min, followed by a linear ramp up to 95% acetonitrile over a period of 14 min. Data acquisition and processing were done using LabSolutions (Shimadzu) and OriginPro (OriginLab®, version 10.1.5.132).

1.2.2. *In vitro* droplet formation

Peptide (25 mM) and ATP (225 mM) stock solutions in DPBS were adjusted to pH 7.4 using a Mettler-Toledo MP220 pH meter after which they were stored at 4 °C and −20 °C (aliquoted), respectively. PolyU (45 mM) stocks were dissolved in nuclease-free water, aliquoted (stored at -20°C) and thawed freshly prior usage. Droplet formation was induced by addition of either ATP or polyU in the presence of peptides. In negative control samples, the volume corresponding to the addition of counterions was replaced by pure DPBS.

1.2.3. Turbidity measurements

Turbidity was assessed on a 384 flat transparent UV-star well plate (Greiner) by measuring the turbidity at 600 nm on a TECAN Spark 20M microplate reader. The data was processed using Excel by Microsoft Corporation and plotted using Origin Pro by OriginLab® 10.1.5.132.

1.2.4. Pull-down measurements

Pull-down experiments were conducted by mixing 1.48 mM of **Fmoc-R_3^{TPP}** + 20 µM **Coum-R_3^{TPP}** + 16 mM of ATP as well as 0.23 mM of **Fmoc-R_3^{TPP}** + 20 µM **Coum-R_3^{TPP}** with 1 mM of polyU. After induction of complex coacervation, samples were centrifuged at 16.2k g for 3 min after which the supernatant was analyzed by TECAN Spark 20M microplate reader on a 384 flat black (Greiner) well plate (λ_{ex} = 488 nm, λ_{em} = 501 nm). The partitioning efficiency was calculated by referencing the fluorescence signal against pure 20 µM **Coum-R_3^{TPP}**. The data was processed using Excel by Microsoft Corporation and plotted using Origin Pro by OriginLab® 10.1.5.132.

1.2.5. Isothermal Titration Calorimetry (ITC)

ITC titrations were performed on a MicroCal PEAQ-ITC instrument from Malvern Panalytical. All experiments were performed at 25°C and with a control titration of the respective peptide

concentration in DPBS buffer (pH 7.4) into the same buffer without any polyanion. All samples (cell and titration solutions) were prepared with the same concentration of DPBS buffer (at pH 7.4) to avoid additional dilution effects. The data was analyzed using a non-linear least squares algorithm provided by the PEAQ-ITC analysis software. The component concentrations were chosen in a regime where little to no coacervation could be observed, for the peptide being 1.67 mM (pU) and 10 mM (ATP) in the syringe and 500 µM (pU) and 1.25 mM (ATP) polyanion monomer concentration in the cell. A titration of 25 injections of 1.5 µL was chosen. In order to take multiple binding sites N into consideration, charges/monomer were calculated and measured. Correspondingly, the ratios given in the integrated heat plot are of the respective charges. The data was processed using Excel by Microsoft Corporation and plotted using Origin Pro by OriginLab® 10.1.5.132.

1.2.6. Dynamic Light Scattering (DLS)

The hydrodynamic radius (R_h) of the coacervates was determined by dynamic light scattering (DLS) on a Zetasizer Nano instrument (Malvern Instruments Ltd., Malvern, UK). Measurements were carried out in UV cuvettes (BRAND®, Wertheim, Germany) with a total sample volume of 80 µL. The instrument was equilibrated at 25 °C before measurements. Each measurement consisted of 11 runs with a run duration of 10 s. Data were analyzed using Zetasizer Software (Malvern Instruments), Excel by Microsoft Corporation and plotted using Origin Pro by OriginLab® 10.1.5.132.

1.2.7. *In vitro* Confocal-laser scanning microscopy (CLSM)

Droplets were prepared in a homemade microreactor water-in oil emulsion set-up as described previously.[1] In brief, the oil phase was prepared by mixing a fluorosurfactant (dSURF, Fluigent, Le KremlinBicêtre, France), supplied as a 2% (v/v) stock in Novec™ 7500 oil, with fluorinated oil (dOIL, Novec™ 7500, Fluigent) to obtain a final surfactant concentration of 1.5% (v/v). 5 µL of the aqueous (droplet containing) sample was added to 50 µL of the oil phase. Microreactors were created by snipping against the Eppendorf® tube. From this emulsion, 40 µL were pipetted below a coverslip glued on a glass slide. For improved visualization, **Fmoc-R_3^{TPP}/**polyU droplets were doped with 8 v/v% of **Coum-R_3^{TPP}** and sequential images acquired by a *STELLARIS 8 Leica DMi8* microscope (*Leica Microsystems*) (λ_{ex} = 440 nm, λ_{em} = 460 nm – 519 nm), a 40x glycerol immersion objective and an Airy unit of 1 were applied. For partitioning experiments, Sulforhodamine B (5 µM stock solution in MQ) or the Chymotrypsin/Chymotrypsin-FITC mixture (200 µM stock solution in MQ) were added to pre-formed droplets formed by 250 µM **Fmoc-R_3^{TPP}**+ 1 mM of polyU to afford a final concentration of 500 nM or 10 µM, respectively. Partitioning was traced by CLSM using λ_{ex} = 561 nm, λ_{em} = 580 nm – 650 nm for Sulforhodamine B λ_{ex} = 488 nm, λ_{em} = 500 nm – 540 nm for Chymotrypsin-FITC, respectively. Images were processed by *LAS X* from *Leica* and scale bars were added in *Fiji ImageJ*.

1.2.8. Fluorescence Recovery After Photobleaching (FRAP)

In vitro

The **Fmoc-R_3^{TPP}/Coum-R_3^{TPP}** system was prepared at a total concentration of 250 µM using 20 µM of **Coum-R_3^{TPP}** and 230 µM of **Fmoc-R_3^{TPP}** in the microreactors. Fluorescence recovery after photobleaching (FRAP) was performed on a Leica TCS SP5 confocal microscope using a 63x/1.2

NA water-immersion objective (λ_{ex} = 488 nm, λ_{em} = 500–550 nm). A circular region of interest (ROI1) of 1.552 μm^2 was photobleached using a brief high-intensity 488 nm laser pulse (100% laser power for ~200–500 ms), and fluorescence recovery was subsequently monitored under low-intensity imaging conditions (5% laser power) over a total duration of 124 s. Image alignment and drift correction were performed using the StackReg and Translation plugins in Fiji. ROIs were defined as follows: ROI1 (bleached area; 1.552 μm^2), ROI2 (entire droplet), and ROI3 (background region). Recovery data were normalized to pre-bleach intensity levels analyzed by EasyFRAP. An intensity offset correction was applied prior to fitting the recovery curves with a double-term exponential model. The data was processed using Excel by Microsoft Corporation and plotted using Origin Pro by OriginLab® 10.1.5.132.

In cellulo

For in-cell FRAP measurements, cells were treated with 480 µM of **Fmoc-R_3^{TPP}** and 20 µM **Coum-R_3^{TPP}** (in FBS-free DMEM) under (i) substrate-depleted or (ii) steady-state conditions. In (i), the medium was exchanged after 1 min of treatment and FRAP experiments were conducted as described for the *in vitro* section. For (ii), no medium was exchanged. For cycled treatment, one droplet was bleached up to five times to generate cycled, periodic responsive behaviour. Image processing was done in Fiji without bleachpoint correction and the data was processed using Excel by Microsoft Corporation and plotted using Origin Pro by OriginLab® 10.1.5.132.

1.2.9. *In cellulo* CLSM

1.2.9.1. Live Cell Imaging

Cellular uptake studies were performed using A549 cells seeded in µ-Slide 8 well-plates (Ibidi GmbH, Germany) at a density of 20 000 cells per well in Dulbecco's Modified Eagle Medium (DMEM) medium containing 10% fetal bovine serum (FBS). After 24 hours, cells were treated with peptide formulations at total peptide concentrations of 125, 250 or 500 µM, containing 105, 230, and 480 µM (respectively) of **Fmoc-R_3^{TPP}** supplemented with 20 µM **Coum-R_3^{TPP}** each for visualization. Stock peptide solutions (in DPBS, pH = 7.4) were diluted with FBS-free medium prior addition to cells. Negative control peptides were supplemented with the same amount of **Coum-R_3^{TPP}** and treated in accordance. In negative controls, samples were treated with FBS-free DMEM medium. Images were acquired by a *STELLARIS 8 Leica DMi8* microscope (*Leica Microsystems*) (λ_{ex} = 440 nm, λ_{em} = 460 nm – 519 nm), a 40x glycerol immersion objective and an Airy unit of 1 were applied. Images were processed by *LAS X* from *Leica* and scale bars were added in *Fiji ImageJ*.

1.2.9.2. Co-stains

Visualization of the mitochondria and the nucleolus was achieved by co-staining with MitoTracker™ Deep Red (λ_{ex} = 641 nm, λ_{em} = 680 nm – 750 nm) or SYTO™ 14 (λ_{ex} = 521 nm, λ_{em} = 532 nm – 598 nm), respectively. Cells were treated for 10 min with either of these stains or in combination prior imaging. Both MitoTracker™ Deep Red and SYTO™ 14 were added as a 1:1333 v/v dilution. Propidium iodide (PI) (λ_{ex} = 561 nm, λ_{em} = 580 nm – 650 nm) was added 1:167 v/v and measured directly after addition. Images were acquired by a *STELLARIS 8 Leica DMi8* microscope (*Leica Microsystems*) with Coumarin 343 signal detection at λ_{ex} = 440 nm, λ_{em} = 460 nm – 519 nm employing a 40x glycerol immersion objective and an Airy unit of 1. For images not taken in the

presence of co-stain SYTO™ 14, Coumarin 343 signal was detected at λ_{ex} = 492 nm, λ_{em} = 497 nm – 537 nm. Images were processed by *LAS X* from *Leica* and scale bars were added in *Fiji ImageJ*.

1.2.9.3. Imaging of fixed samples

Cells were washed twice with PBS after which they were fixed immediately by pre-warmed paraformaldehyde in PBS (4% Roti-Histofix) for 8 min at 37°C. Afterwards, the cells were washed again twice with PBS. For phalloidin staining, cells were permeabilized by treatment for 5 min at RT using 0.1% Triton-X in PBS supplemented with 1% BSA after which the cells were washed twice with PBS. Phalloidin-555 was prepared as a 1 µL/mL solution in PBS and cells were treated with 240 µL for 45 min. Cells were again washed twice with PBS prior imaging. Images were acquired by a *STELLARIS 8 Leica DMi8* microscope (*Leica Microsystems*) using λ_{ex} = 561 nm, λ_{em} = 570 nm – 620 nm for phalloidin-555, λ_{ex} = 492 nm, λ_{em} = 497 nm – 537 nm for the Coumarin 343 channel and λ_{ex} = 641 nm, λ_{em} = 652 nm – 746 nm for MitoTracker™ Deep Red. A 40x glycerol immersion objective and an Airy unit of 1 were applied. Images were processed by *LAS X* from *Leica* and scale bars were added in *Fiji ImageJ*.

1.2.10. Resazurin-based cell metabolism assay

Metabolic activity was quantified using a resazurin-based assay (Sigma Aldrich, TOX8) in black half area 96-well plates according to the manufacturer's instructions. 3200 Cells were seeded and grown in 100 µL FBS containing DMEM medium for 24 hours. The cells were then treated with the peptides in FBS-free DMEM and 10 % resazurin. Fluorescence was recorded on a SPARK 20M microplate reader (Tecan Group Ltd) with an excitation wavelength of 560 nm and an emission wavelength of 590 nm every hour until 9 hours of incubation. The data was processed using Excel by Microsoft Corporation and plotted using Origin Pro by OriginLab® 10.1.5.132.

1.2.11. Cell viability assay

Cell viability was quantified using the CellTiter-Glo® Luminescent Cell Viability Assay (Promega) in half-area, white 96-well plates according to the manufacturer's instructions. 3200 Cells were seeded and grown in 50 µL FBS containing medium and incubated for 24 hours. The cells were then treated with the peptides in FBS-free DMEM (50 µL total volume). The cells were incubated with 500 µM of **Fmoc-R_3^{TPP}** for 1 min before medium exchange. No medium exchange was done for the 500 µM **Fmoc-R_3^{TPP}**-treated positive, and DMEM-treated negative controls. CellTiter-Glo® Luminescent Cell Viability Assay was performed according to the manufacturer's instructions and luminescence was recorded on a SPARK 20M microplate reader (Tecan Group Ltd) after 24 hours of incubation. The data was processed using Excel by Microsoft Corporation and plotted using Origin Pro by OriginLab® 10.1.5.132.

1.2.12. Extracellular flux experiments

A549 cells were seeded into a Seahorse XFe96/XF Pro Cell Culture Microplate with 10 000 cells per well in Dulbecco's Modified Eagle's Medium (DMEM) with 10% FBS and incubated until the next day at 37 °C and 5 % CO_2. Additionally, a XFe96/XF Pro sensor cartridge was hydrated in 200

µL Seahorse XF Calibrant o.n. at 37 °C. After 24 h, XF Medium was prepared by supplementing Seahorse XF DMEM pH 7.4 with 1 mM pyruvate, 2 mM glutamine and 10 mM glucose. Growth medium (DMEM, 10% FBS) was exchanged by assay medium (supplemented XF DMEM) and then incubated for 45-60 minutes at 37 °C. In the meantime, Seahorse XF Cell Mito Stress Test Kit was prepared. Therefore, assay compounds were solubilized in XF DMEM: Oligomycin was resuspended in 630 µL medium, FCCP in 720 µL and Rot/AA in 540 µL medium. These stocks were then diluted into final concentrations of 1.5 µM Oligomycin, 1 µM FCCP and 0.5 µM Rot/AA. The assay compounds were loaded into the cartridge first before preparing the samples. Samples were prepared in XF Medium with a final PBS concentration of 2 vol%. After samples were loaded into the cartridge, XFe96/XF Pro sensor cartridge and XFe96/XF Pro Cell Culture Microplate were placed into the Seahorse XFe96 Analyzer and start the measurement for 12 h.

1.2.13. Annexin-V staining

For Annexin V/PI-based cell apoptosis assay, cells were seeded in a µ-Slide 8 well-plates (Ibidi GmbH, Germany) at a density of 20 000 cells per well in Dulbecco's Modified Eagle Medium (DMEM) medium containing 10% FBS. After 24 hours, cells were treated with 500 µM **Fmoc-R$_3^{TPP}$** in FBS-free DMEM for either 1 min or 1 h. Negative controls were treated with only DMEM. After washing once with cold PBS, cells were treated with Annexin V-FITC (2.5 vol%) and PI (5 vol%) in binding buffer included in the MCE Apoptosis detection kit. After 10 min at RT, cells were imaged using a *STELLARIS 8 Leica DMi8* microscope (*Leica Microsystems*) with λ_{ex} = 488 nm, λ_{em} = 526 nm – 571 nm for Annexin V-FITC and λ_{ex} = 535 nm, λ_{em} = 599 nm – 650 nm for PI.

1.2.14. Apoptosis array

Cells were seeded in 6-well plates at a density of 1.92 x 10^5 cells per well in 3 mL of medium supplemented with fetal bovine serum (FBS). After 18 h, the medium was replaced with 3 mL of serum-free medium containing 500 µM of **Fmoc-R$_3^{TPP}$**. Following the incubation time (of either 1 min or 1 h), the cells were washed twice with phosphate-buffered saline (PBS) and treated with Lysis Buffer 17 (250 µL per well; R&D Systems) for 30 min at 4°C. The resulting lysates were centrifuged at 14,000 × g for 15 min at 4°C to remove cellular debris. The supernatants were analyzed using the Human Apoptosis Array (R&D Systems, ARY007) according to the manufacturer's protocol. Briefly, the samples were diluted and incubated with a cocktail of biotinylated detection antibodies. The mixtures were then incubated with the nitrocellulose membranes containing immobilized capture antibodies. After washing to remove unbound proteins, the membranes were treated with Streptavidin-HRP followed by chemiluminescent detection reagents. Signal intensity was captured using a ChemiDoc Imaging System (Bio-Rad). Protein expression was quantified by calculating the background-subtracted mean pixel density of duplicate spots using the "Gels" plugin in ImageJ software. The data was processed using Excel by Microsoft Corporation and plotted using Origin Pro by OriginLab® 10.1.5.132.

1.2.15. Cell lysate binding assay

A549 cells were incubated at a quantity of 100000 cells per well in 1 ml DMEM supplemented with 10% FBS in a 6-well cell culture multiwell plate to adhere for 24 hours. Following 24-hour attachment, cells were washed with PBS with gentle rocking. The PBS was decanted and replaced with ice-cold RIPA buffer (Serva Electrophoresis). Cells incubated in lysis buffer for 10 min on ice, to be scraped off the culture dish using a cell scraper. The cell suspension was gently transferred to a pre-cooled micro-centrifuge tube and slowly agitated for 30 minutes at 4°C, then clarified by spinning in a micro-centrifuge tube for 10 min at 13000 rpm and 4°C. The supernatant was gently collected and stored at -20°C. Turbidity was measured as described previously by measuring either cell lysate alone or in the presence of 500 μM **Fmoc-R_3^{TPP}**.

1.3. Synthesis and purification

1.3.1. Synthesis of short cationic peptides

Peptides were synthesized using the Fmoc-based solid-phase peptide synthesis (Fmoc-SPPS) strategy, synthesizing the sequence from the C- to the N-terminus in a heat-assisted setup under nitrogen flow. Rink amide MBHA resin was used at scales of 0.25–0.5 mmol. Prior to synthesis, the resin was swollen in DMF at room temperature for 1 h and subsequently transferred to the synthesis setup, after which the solvent was removed by vacuum-assisted filtration. Before each coupling step, the Fmoc protecting group was removed by two consecutive deprotection steps using 5% piperazine in DMF (10 mL per 0.25 mmol scale), applied for 1 min followed by 5 min at 68 °C. The resin was then filtered and washed four times with DMF (30 s each).

Amino acids (0.2 M in DMF) were coupled twice using O-(1H-6-chlorobenzotriazol-1-yl)-1,1,3,3-tetramethyluronium hexafluorophosphate (HCTU, 0.4 M in DMF) and N,N'-diisopropylethylamine (DIPEA, 0.4 M in DMF), with heating at 68 °C for 5 min per coupling. After each coupling, the reaction mixture was filtered and the resin washed twice with DMF (30 s). Subsequent coupling cycles were performed analogously. For introducing the triphenylphosphate (TPP) moiety on the lysine side chain, Fmoc-Lys(Mtt)-OH was employed and its side chain selectively deprotected with DCM, 1% TFA and 5% TIPS as described previously and displayed in supplementary scheme 1.[2] Coupling of TPP to the Lys side chain was performed as outlined for amino acid couplings, but was coupled four instead of two times. For **Coum-R$_3$^{TPP}**, Coumarin 343 was coupled overnight using 3 equivalents of PyBOP, 1.2 equivalents of Coumarin 343 and 6 equivalents of DIPEA at RT. For **Acet-R$_3$^{TPP}**, the N-terminus was capped after final Fmoc deprotection applying 6 equivalents of acetic anhydride and 6 equivalents of DIPEA twice for 10 min at RT.

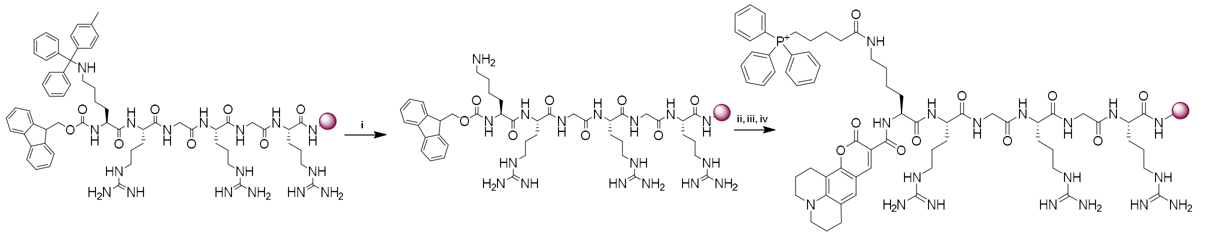

Scheme S 1: Synthesis of TPP peptide derivatives by SPPS on rink amide MBHA resin. (**i**) 1 % TFA, 5 % TIPS in DCM, RT, 20-30 x 2.5 min (**ii**) 3 equ. (4-carboxybutyl)triphenylphosphonium bromide, 6 equ. HCTU, 6 equ. N,N'-DIPEA in DMF, 68 °C, 4 x 6 min (**iii**) 5 wt% Piperazine in DMF, 68 °C, 2 x 6 min (**iv**) 3 equ. PyBOP, 1.2 equ. Coumarin 343, 6 equ. DIPEA, RT, o.n. The synthesis of acetylated and Fmoc versions were done in accordance and is described in detail in the synthesis section.

1.3.2. Purification of peptides

The resin was treated with a cleavage cocktail consisting of TFA/TIPS/H_2O (95:2.5:2.5, v/v/v) for 2.5 hours. After filtration, the resin was washed with TFA (1 mL). The crude peptide was precipitated by addition of cold diethyl ether (60 mL), followed by centrifugation (4000 rpm, 0 °C, 20 min), and the supernatant was decanted. The crude material was dissolved in ACN/H_2O (9:1, v/v, containing 5% TFA) to a total volume of 20 mL and filtered through a 0.2 µm syringe filter.

Purification was performed by reversed-phase HPLC using a Phenomenex Gemini NX-C18 column (5 µm, 110 Å, 150 × 30 mm) at a flow rate of 25 mL/min. The gradient elution started at 0% ACN (0.1% TFA in water) and was linearly increased to 100% ACN (0.1% TFA). After solvent removal under high vacuum, the identity of the compounds was confirmed by LC–MS.

2. Supplementary data

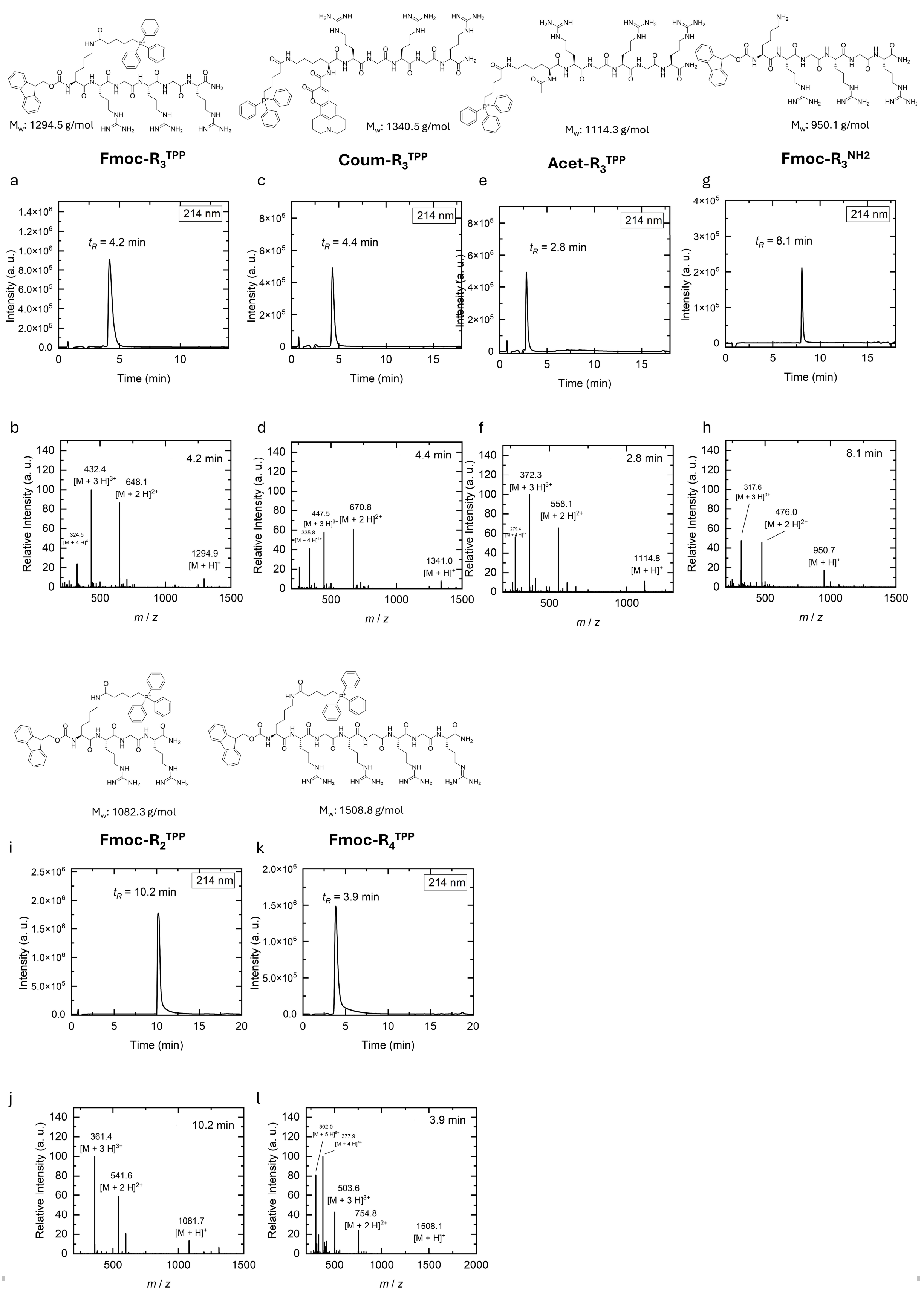

Mw: 1294.5 g/mol
Fmoc-R3TPP
Mw: 1340.5 g/mol
Coum-R3TPP
Mw: 1114.3 g/mol
Acet-R3TPP
Mw: 950.1 g/mol
Fmoc-R3NH2
a
214 nm
tR = 4.2 min
Intensity (a. u.)
Time (min)
c
214 nm
tR = 4.4 min
e
214 nm
tR = 2.8 min
g
214 nm
tR = 8.1 min
b
4.2 min
432.4
[M + 3 H]3+
648.1
[M + 2 H]2+
324.5
[M + 4 H]4+
1294.9
[M + H]+
Relative Intensity (a. u.)
m / z
d
4.4 min
447.5
[M + 3 H]3+
670.8
[M + 2 H]2+
335.8
[M + 4 H]4+
1341.0
[M + H]+
f
2.8 min
372.3
[M + 3 H]3+
558.1
[M + 2 H]2+
279.4
[M + 4 H]4+
1114.8
[M + H]+
h
8.1 min
317.6
[M + 3 H]3+
476.0
[M + 2 H]2+
950.7
[M + H]+
Mw: 1082.3 g/mol
Fmoc-R2TPP
Mw: 1508.8 g/mol
Fmoc-R4TPP
i
214 nm
tR = 10.2 min
k
214 nm
tR = 3.9 min
j
10.2 min
361.4
[M + 3 H]3+
541.6
[M + 2 H]2+
1081.7
[M + H]+
l
3.9 min
302.5
[M + 5 H]5+
377.9
[M + 4 H]4+
503.6
[M + 3 H]3+
754.8
[M + 2 H]2+
1508.1
[M + H]+

Figure S1: Purity characterization of short cationic peptides. **a**, LC elugram of Fmoc-K(TPP)RGRGR-$CONH_2$ (**Fmoc-R_3^{TPP}**). **b**, Mass spectrum of **Fmoc-R_3^{TPP}**. **c**, LC elugram of Coumarine343-K(TPP)RGRGR-$CONH_2$ (**Coum-R_3^{TPP}**). **d**, Mass spectrum of **Coum-R_3^{TPP}**. **e**, LC elugram of Acetyl-K(TPP)RGRGR-$CONH_2$ (**Acet-R_3^{TPP}**). **f**, Mass spectrum of **Acet-R_3^{TPP}**. **g**, LC elugram of Fmoc-KRGRGR-$CONH_2$ (**Fmoc-R_3^{NH2}**). **h**, Mass spectrum of **Fmoc-R_3^{NH2}**. **i**, LC elugram of Fmoc-K(TPP)RGR-$CONH_2$ (**Fmoc-R_2^{TPP}**). **j**, Mass spectrum of **Fmoc-R_2^{TPP}**. **k**, LC elugram of Fmoc-K(TPP)RGRGRGR-$CONH_2$ (**Fmoc-R_4^{TPP}**). **l**, Mass spectrum of **Fmoc-R_4^{TPP}**.

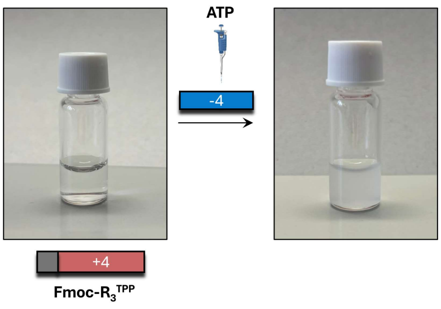

Figure S2: Change in macroscopic property from 1.5 mM **Fmoc-R_3^{TPP}** before (left) and after (right) the addition of 16 mM of ATP. Buffer conditions: DPBS at pH 7.4.

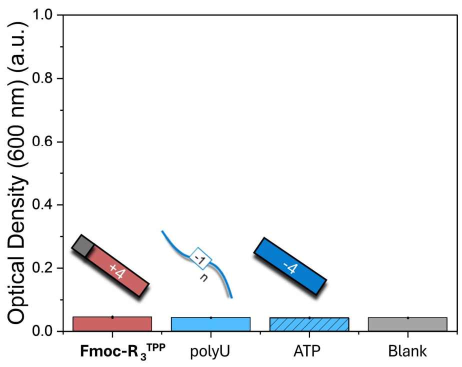

Figure S3: Turbidity measurements of individual, **Fmoc-R_3^{TPP}** (1.5 mM), ATP (16 mM) or polyU (1 mM) at 600 nm. Error bars represent standard deviations from triplicates (n=3). Buffer conditions: DPBS at pH 7.4.

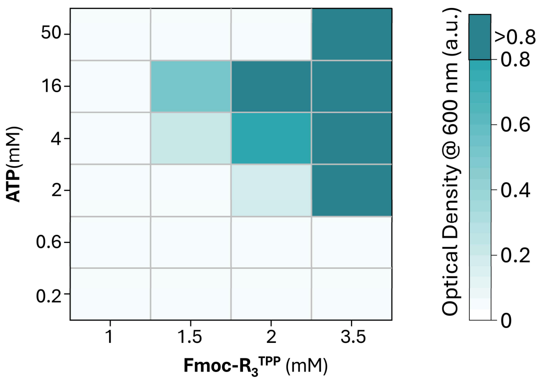

Figure S4: Phase diagram of **Fmoc-R$_3$^{TPP}** when ATP is present as determined by turbidity measurements. Measurements were conducted at 600 nm in triplicates (n=3). In the phase diagram, means are shown as representative data points. Buffer conditions: DPBS at pH 7.4.

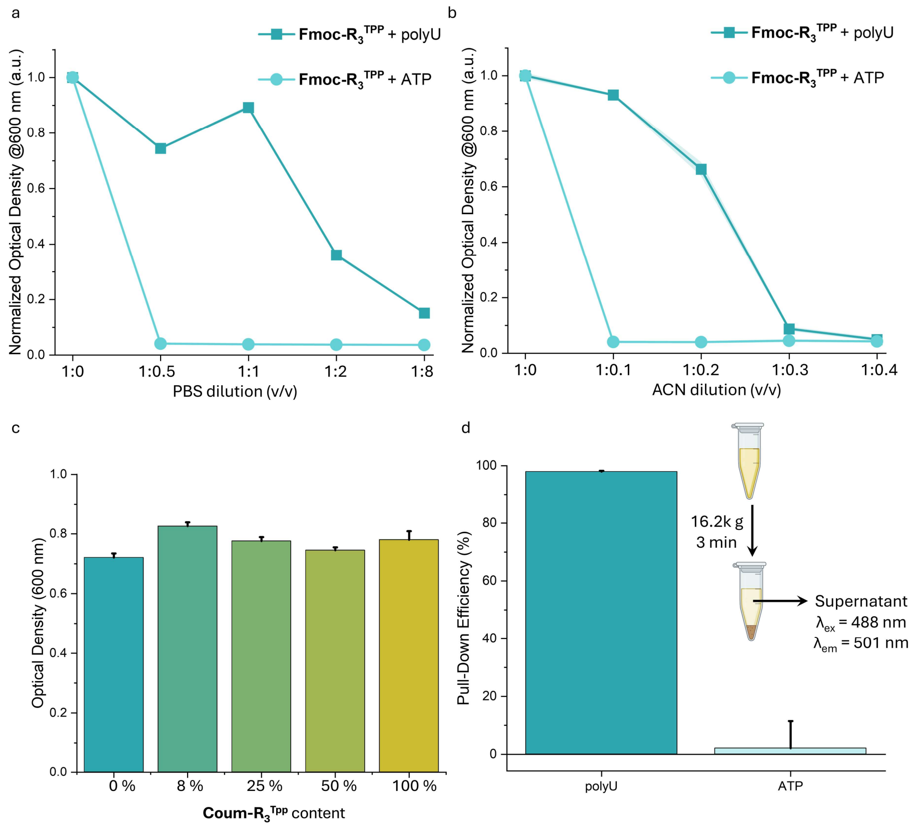

Figure S5: Stability tests of droplets formed by polyU or ATP in the presence of **Fmoc-R$_3$^{TPP}**. **a**, DPBS dilution tests of pre-formed coacervates formed by 250 µM **Fmoc-R$_3$^{TPP}** + 1 mM of polyU or 1.5 mM **Fmoc-R$_3$^{TPP}** + 16 mM ATP. Standard deviations from triplicates (n=3) are too small to visualize. **b**, DPBS dilution tests of pre-formed coacervates formed by 250 µM **Fmoc-R$_3$^{TPP}** + 1 mM of polyU or 1.5 mM **Fmoc-R$_3$^{TPP}** + 16 mM ATP. Standard deviations are shown from triplicates (n=3). **c**, Effect of **Coum-R$_3$^{TPP}** on droplet formation behavior. Turbidity measurements indicate no interference of **Coum-R$_3$^{TPP}** with **Fmoc-R$_3$^{TPP}** /polyU droplets. Measurements were done at 600 nm. Error bars represent standard deviations from triplicates (n=3). **d**, Fluorescent pull-down experiment using 250 µM **Coum/Fmoc-R$_3$^{TPP}** + 1 mM of polyU or 1.5 mM **Coum/ Fmoc-R$_3$^{TPP}** + 16 mM of ATP. After centrifugation, the pull-down efficiency is determined by comparing the supernatant of the samples with a pure 20 µM **Coum-R$_3$^{TPP}** sample. Error bars represent standard deviations of triplicates (n=3). Buffer conditions: DPBS at pH 7.4.

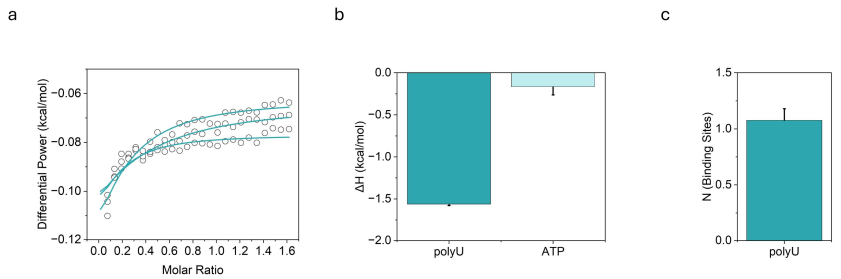

Figure S6: ITC measurements quantifying interaction parameters between **Fmoc-R$_3$^{TPP}** and ATP or polyU. **a**, ITC titration curves from titrating **Fmoc-R$_3$^{TPP}** to ATP. Three curves correspond to n=3 replicas. **b**, Reaction enthalpy as determined by ITC measurements from titrating **Fmoc-R$_3$^{TPP}** to either ATP or polyU. **c**, Stoichiometry between **Fmoc-R$_3$^{TPP}** and polyU. Due to low signal intensity of ATP, the number of binding sites N could not be reliably determined. Error bars represent standard deviations from triplicates (n=3). Buffer conditions: DPBS at pH 7.4.

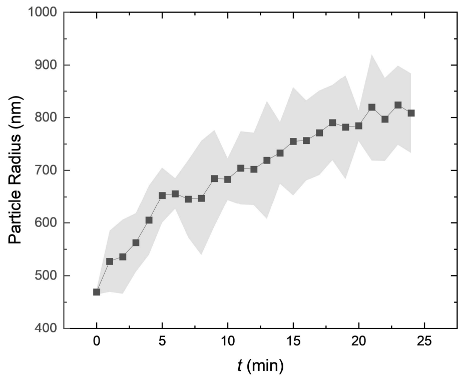

Figure S7: Time-dependent dynamic light scattering of **Fmoc-R$_3$^{TPP}** and polyU. The dynamic behaviour of droplets formed by 250 µM **Fmoc-R$_3$^{TPP}** and 1 mM of polyU in dependence of time. Error bars represent standard deviations from triplicates (n=3). Buffer conditions: DPBS at pH 7.4.

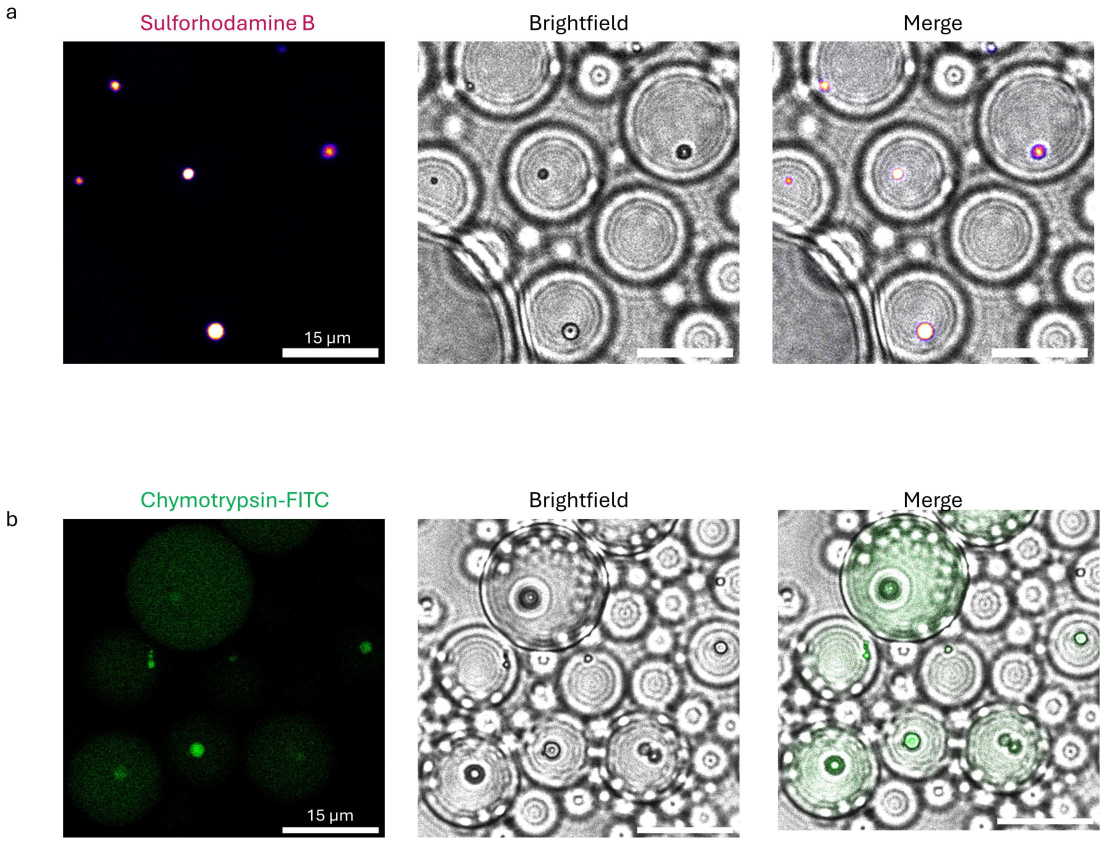

Figure S8: Partitioning experiments into droplets formed by 250 µM **Fmoc-R$_3^{TPP}$** and 1 mM of polyU of (**a**) Sulforhodamine B (500 nM) or (**b**) 5 µM of Chymotrypsin in the presence of 5 µM Chymotrypsin-FITC. Buffer conditions: DPBS at pH 7.4.

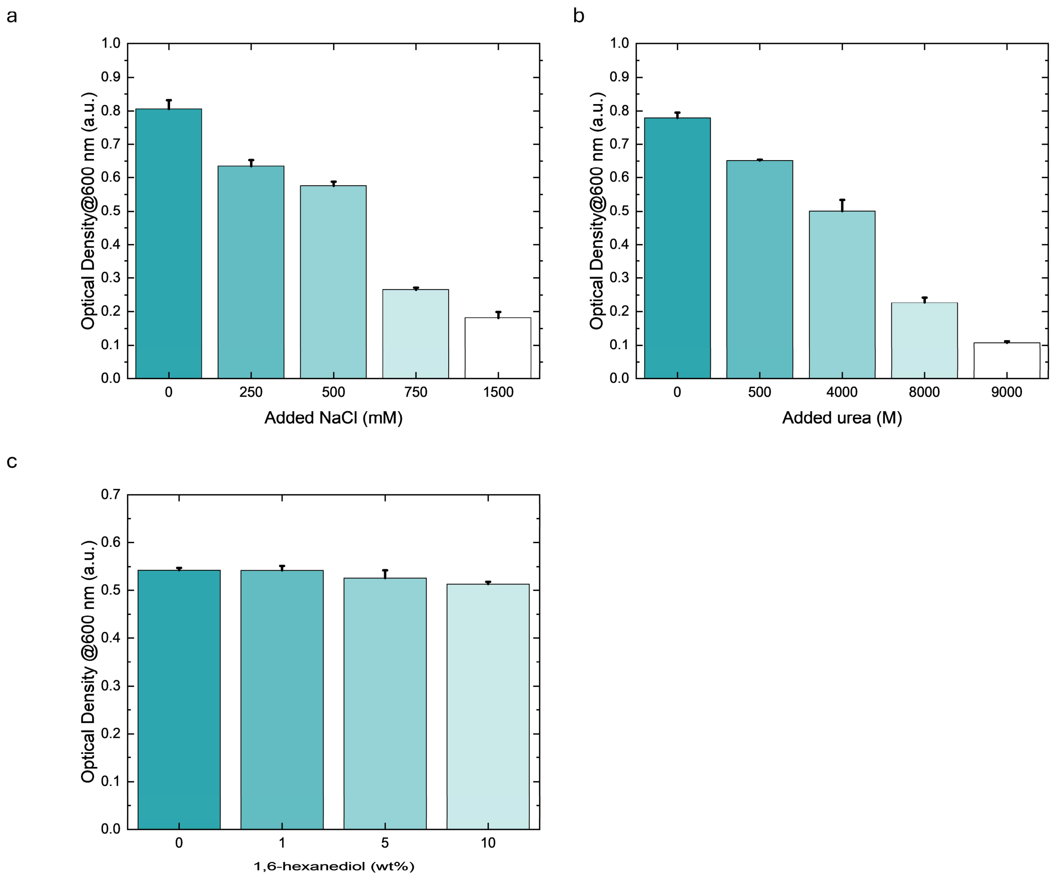

Figure S9: Droplet stability against disturbing molecules. Droplets formed by 250 µM **Fmoc-R$_3^{TPP}$** and 1 mM of polyU were subjected to various amounts of (**a**) NaCl and (**b**) urea and (**c**) 1,6-hexanediol. Error bars represent standard deviations from triplicates (n=3). Buffer conditions: DPBS at pH 7.4.

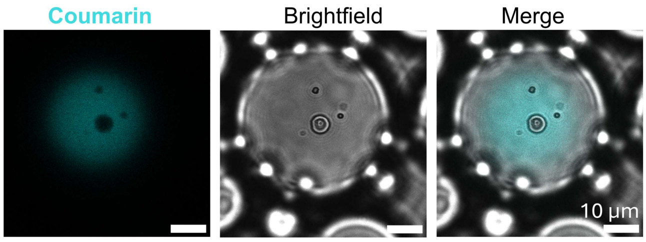

Figure S10: CLSM images of [**Coum-R$_3^{TPP}$**] = 250 µM, [polyU] = 1 mM in DPBS showing self-quenching effect in concentrated droplets.

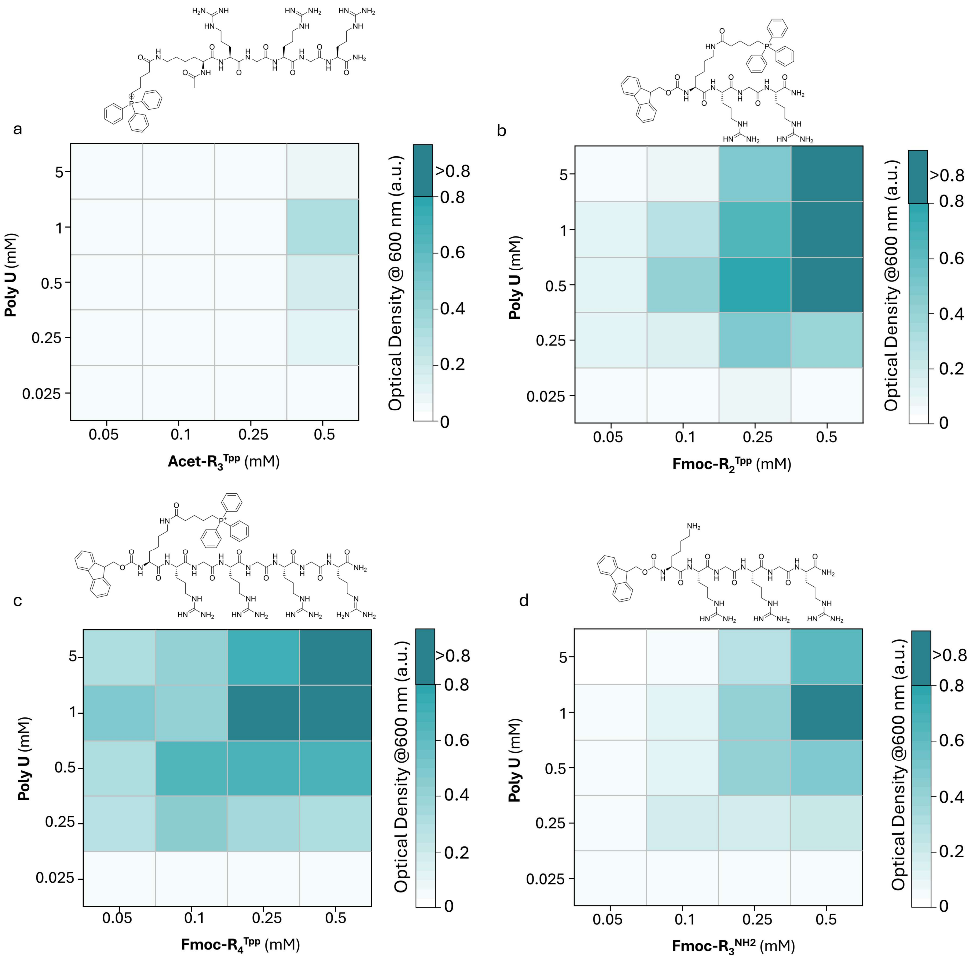

` `

Figure S11: Phase diagrams as determined by turbidity measurements. Varying concentrations of **a**, **Acet-R$_3^{TPP}$**, **b**, **Fmoc-R$_2^{TPP}$**, **c**, **Fmoc-R$_4^{TPP}$**, **d**, **Fmoc-R$_3^{NH2}$** were subjected to varying concentrations of polyU and the optical density measured at 600 nm. Data points correspond to means of n=3 replicas.

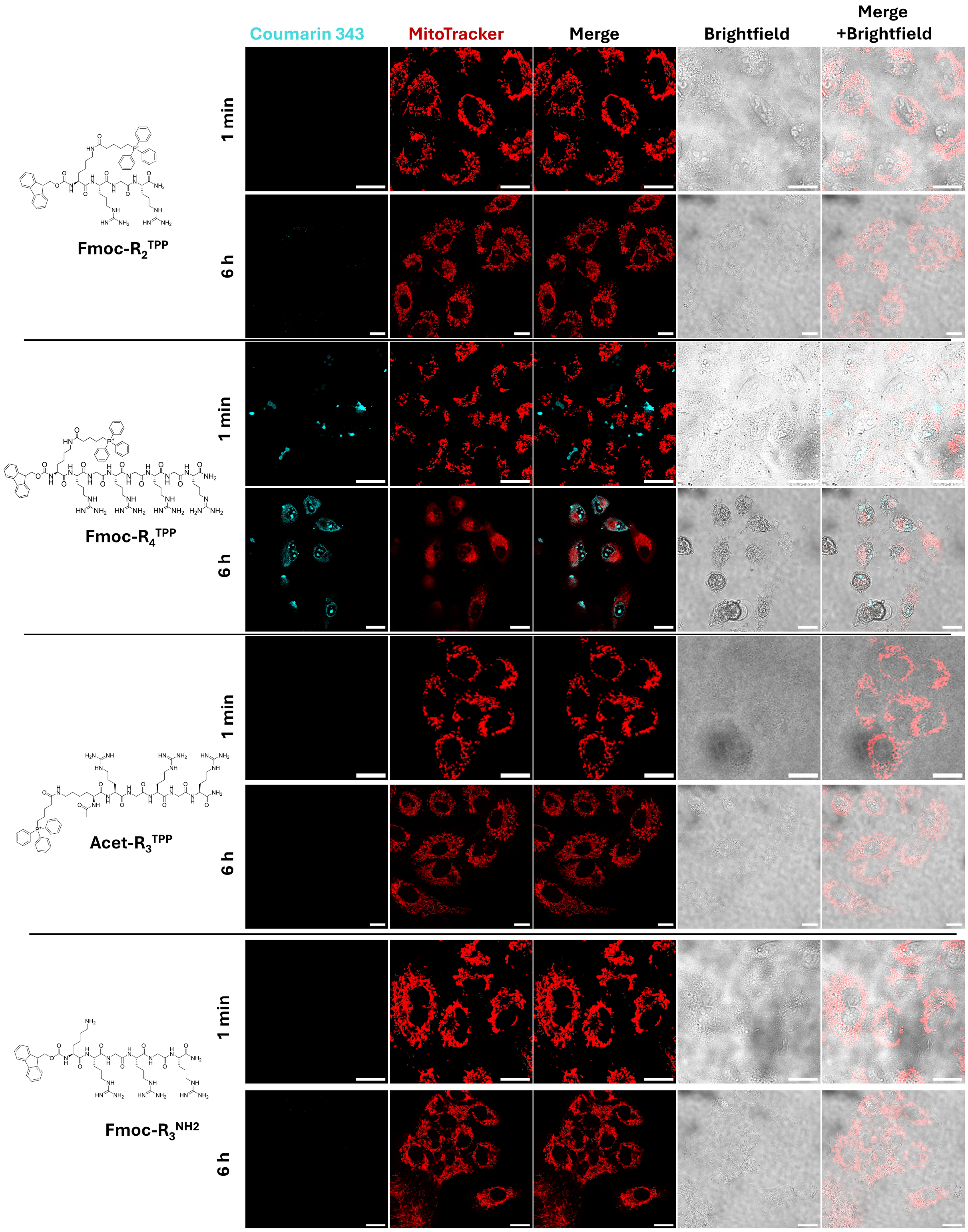

Figure S12: CLSM of live-cell peptide-treated A549 cells. The respective peptides were applied at 480 µM, keeping **Coum-R$_3$^{TPP}** consistent at 20 µM and the cells were incubated for 1 min or 6 hours. For all experiments, FBS-free DMEM media was employed. Scale bars are 20 µm.

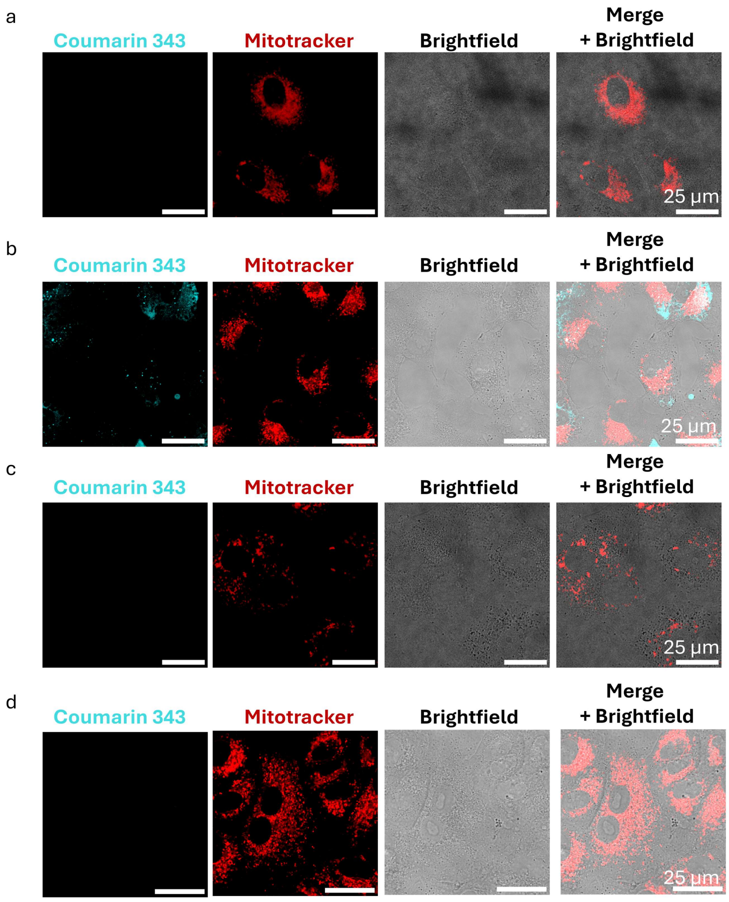

Figure S13: CLSM control experiments. **a**, Living A549 cells treated with [**Coum-R$_3$**^{TPP}] = 20 µM for 6 h. **b**, Fixed A549 cells after 6 h of treatment with [**Coum-R$_3$**^{TPP}] = 20 µM**.** **c**, Living A549 cells treated with DMEM for 6 h. **d**, Fixed A549 cells treated with DMEM for 6 h. For all experiments, FBS-free DMEM medium was employed.

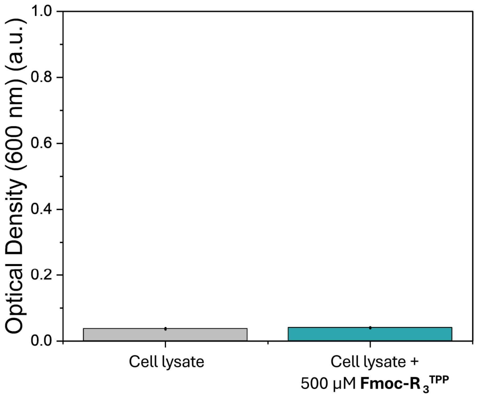

Figure S14: Optical density of cell lysate in the absence and presence of 500 µM of **Fmoc-R$_3^{TPP}$** to determine unspecific binding.

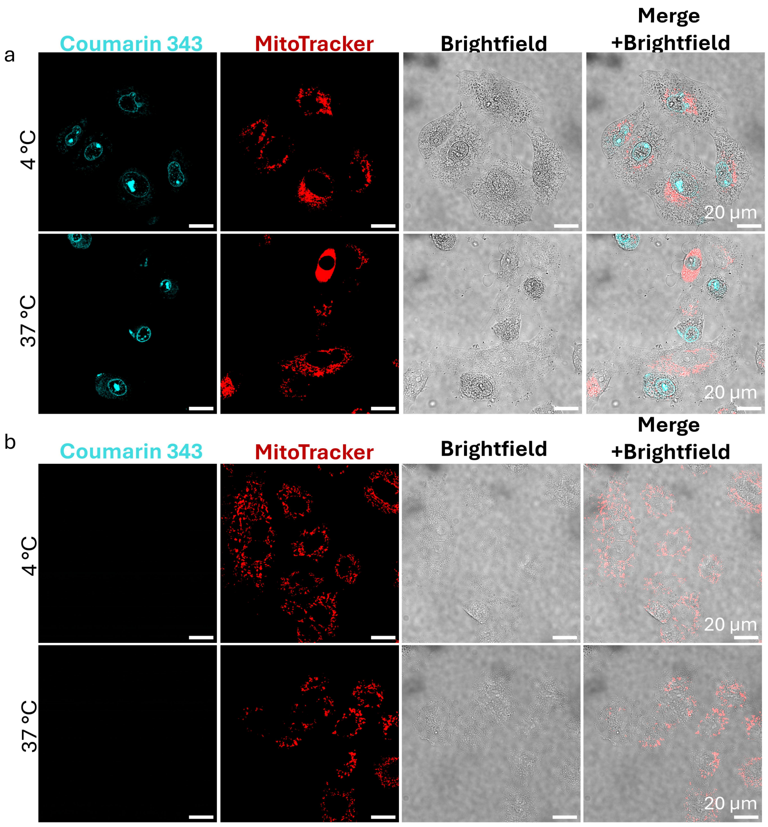

Figure S15: Temperature-dependent peptide uptake studies. **a,** CLSM images of A549 cells treated with 500 µM of **Fmoc-R$_3^{TPP}$/Coum-R$_3^{TPP}$** after 2 hours at 4 °C (top) or 37 °C (bottom). **b,** DMEM-treated A549 controls.

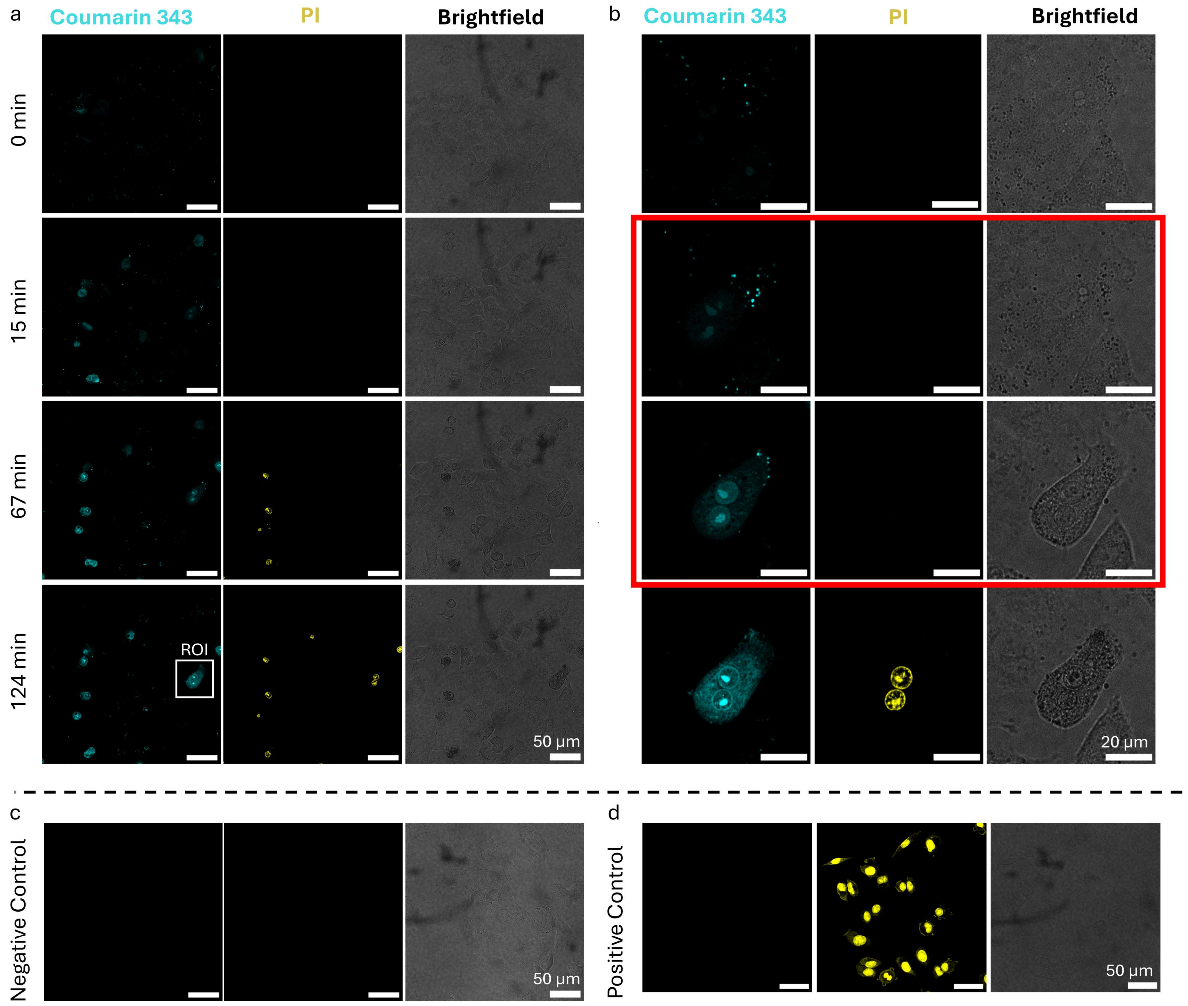

Figure S16: CLSM of **Fmoc-R$_3$^{TPP}/Coum-R$_3$^{TPP}** treated A549 cells to elucidate cellular uptake mechanism in the presence of PI. **a**, Time-dependent CLSM analyses of A549 cells treated with 250 µM of **Fmoc-R$_3$^{TPP}/Coum-R$_3$^{TPP}**. **b**, Zoom of ROI as indicated in A. **c**, A549 cells treated with DMEM. **d**, Positive control pure DMSO-treated A549 cells.

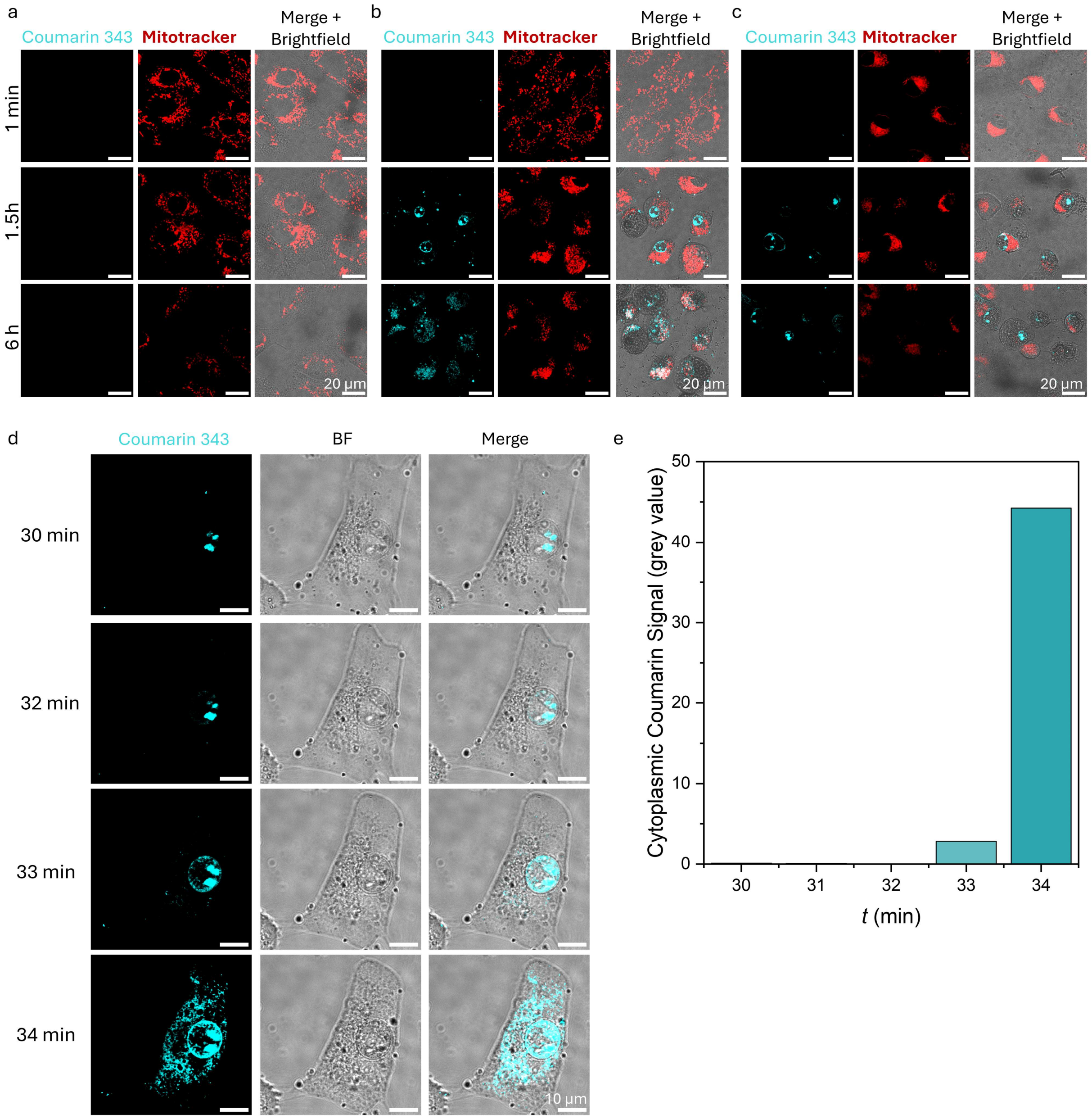

Figure S17: Time-dependent CLSM live-cell imaging of A549 cells subjected to **Fmoc-R$_3$^{TPP}/Coum-R$_3$^{TPP}** system with varying concentrations. **a**, A549 cells treated with [**Fmoc-R$_3$^{TPP}**] = 105 µM + 20 µM **Coum-R$_3$^{TPP}**. **b**, A549 cells treated with [**Fmoc-R$_3$^{TPP}**] = 230 µM + 20 µM **Coum-R$_3$^{TPP}**. **c**, A549 cells treated with [**Fmoc-R$_3$^{TPP}**] = 480 µM + 20 µM **Coum-R$_3$^{TPP}**. **d**, Detailed image series of permeabilized A549 cells when treated with [**Fmoc-R$_3$^{TPP}**] = 480 µM + 20 µM **Coum-R$_3$^{TPP}**. **e**, Quantification of time-dependent series depicted in **d**.

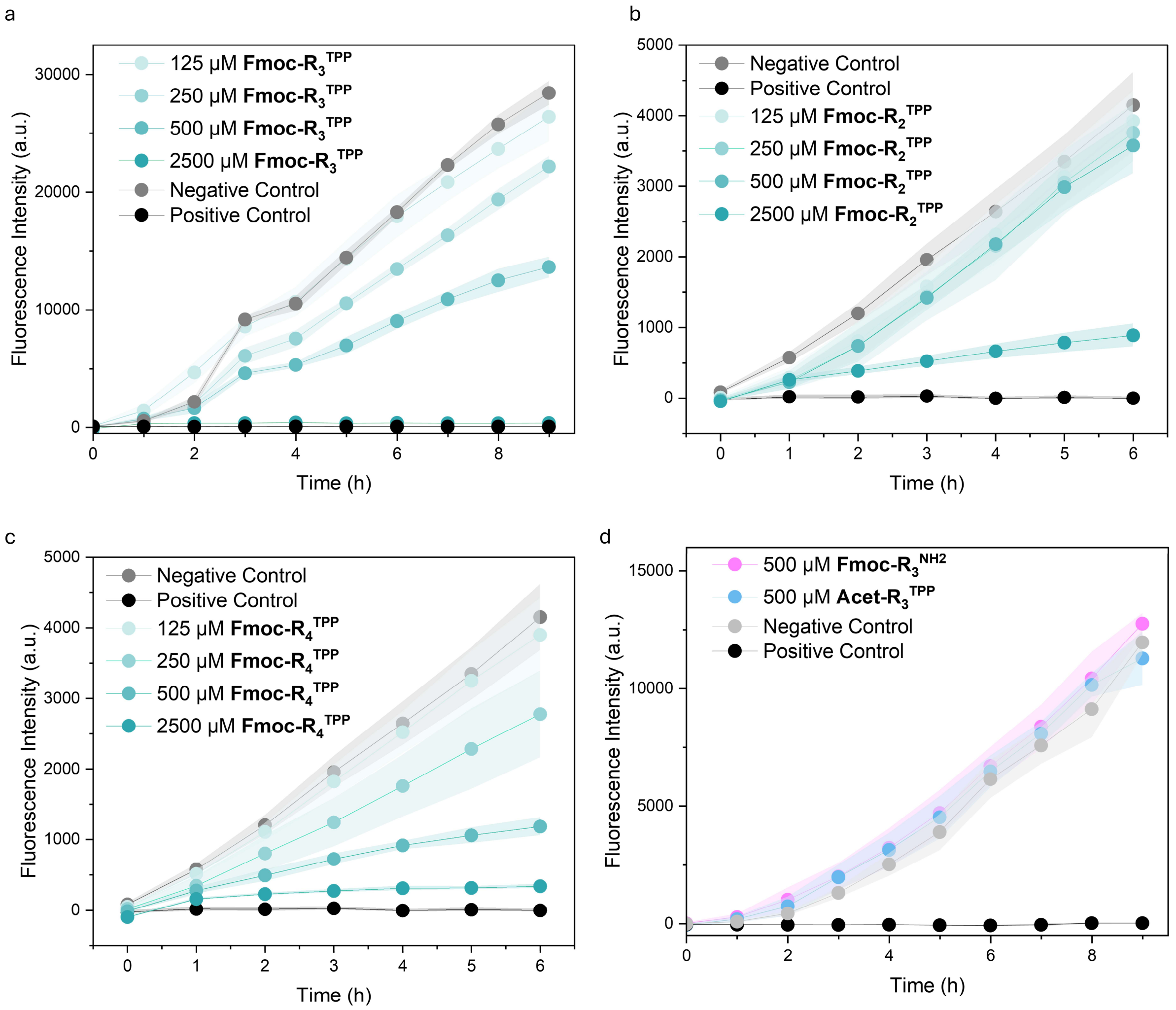

Figure S18: Time-dependent resazurin-based metabolic assay. **a**, A549 cells treated with varying concentration of **Fmoc-R$_3^{TPP}$**. **b**, A549 cells treated with varying concentration of **Fmoc-R$_2^{TPP}$**. **c,** A549 cells treated with varying concentration of **Fmoc-R$_4^{TPP}$**. **d**, A549 cells treated with either **Fmoc-R$_3^{NH2}$**or **Acet-R$_3^{TPP}$**. Error bars represent standard deviations from n=6 replicas. For all experiments, DMEM and pure DMSO were employed as negative and positive control, respectively.

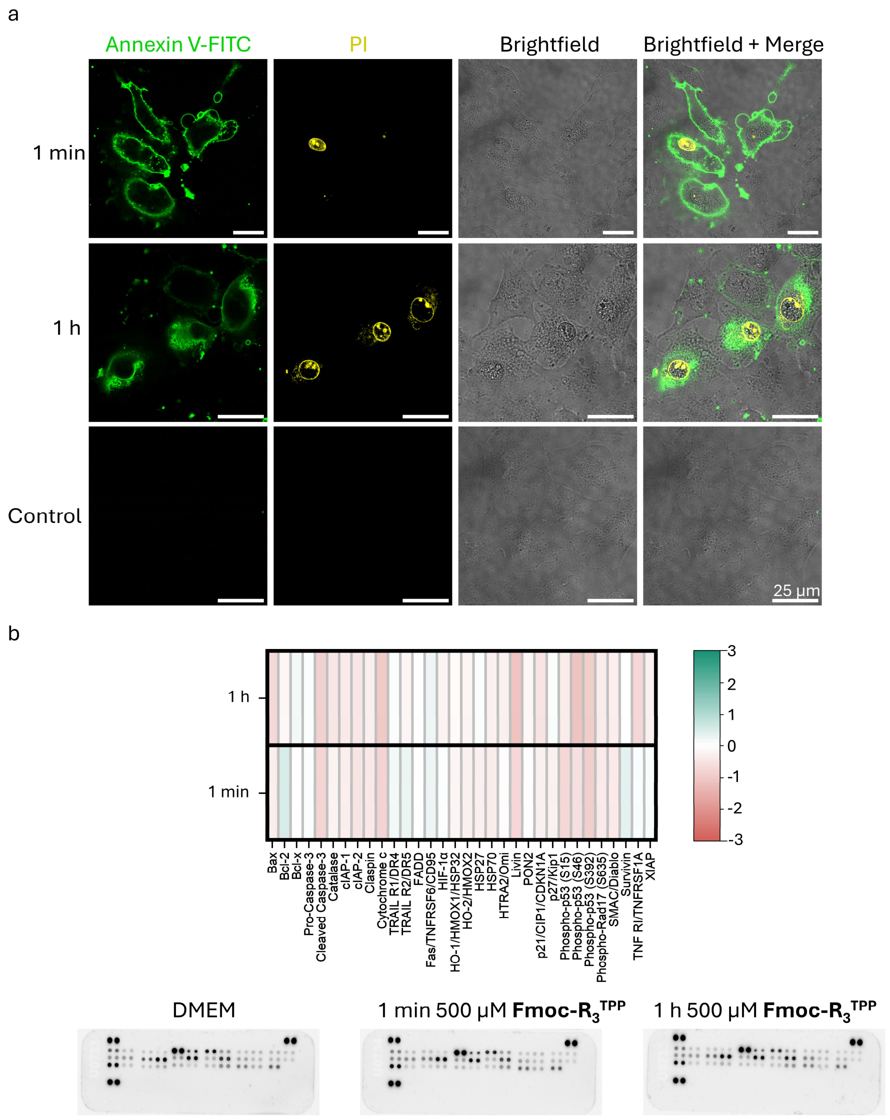

Figure S19: Analyses of cellular decay. **a,** Annexin-V/PI staining. A549 cells were treated with 500 µM **Fmoc-R$_3$^{TPP}** for either 1 min or 1 h after which the cells were stained by Annexin V-FITC and PI. The A549 control was treated with pure DMEM. **b,** Apoptosis protein profiler. A549 cells were treated with 500 µM **Fmoc-R$_3$^{TPP}** for either 1 min or 1 h. Top graph shows log$_2$-fold change, bottom the corresponding proteome analysis array.

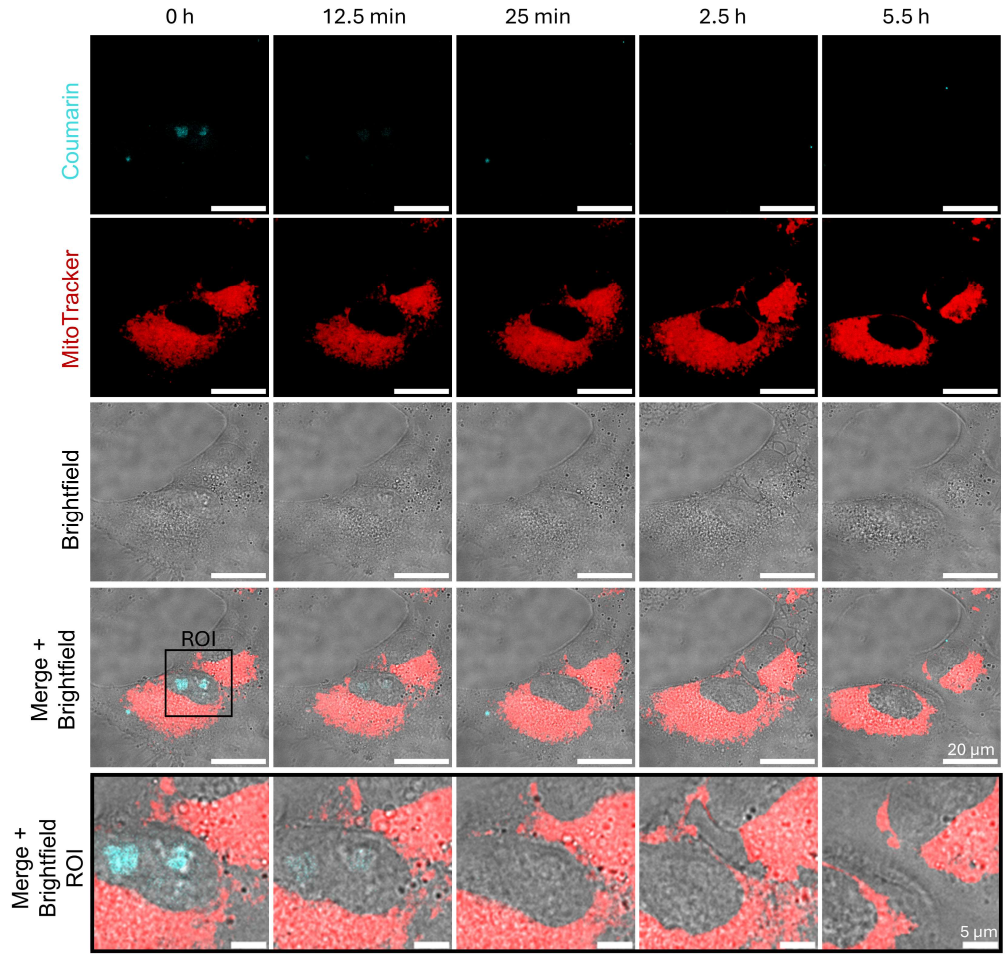

Figure S20: Cell division in CLSM live-cell imaging of recovering **Fmoc-R$_3^{TPP}$** -treated A549 cells. Cells were treated for 1 min with [**Fmoc-R$_3^{TPP}$**] = 480 µM + 20 µM **Coum-R$_3^{TPP}$** after which media was exchanged. Droplet signal disappears after which cells continue dividing.

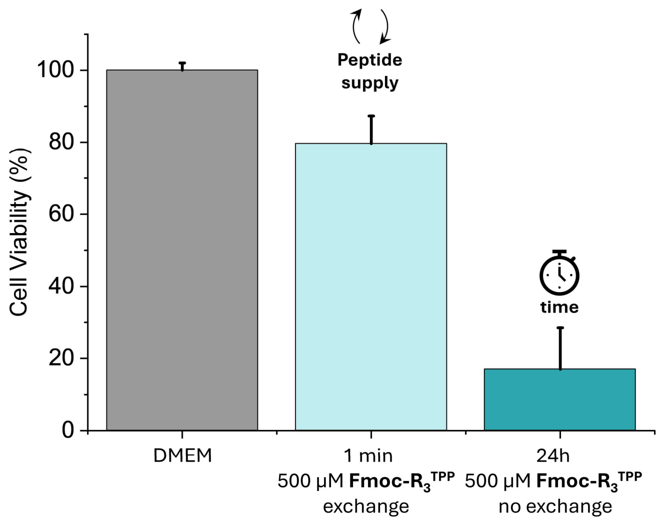

Figure S21: Cell viability assay of recovery experiment. Cells were either treated with DMEM, 500 μM of **Fmoc-R$_3$^{TPP}** for 1 min after which the media was exchanged to DMEM or for 24 hours with 500 μM of **Fmoc-R$_3$^{TPP}**. Error bars represent standard deviations from n=6 replicas.

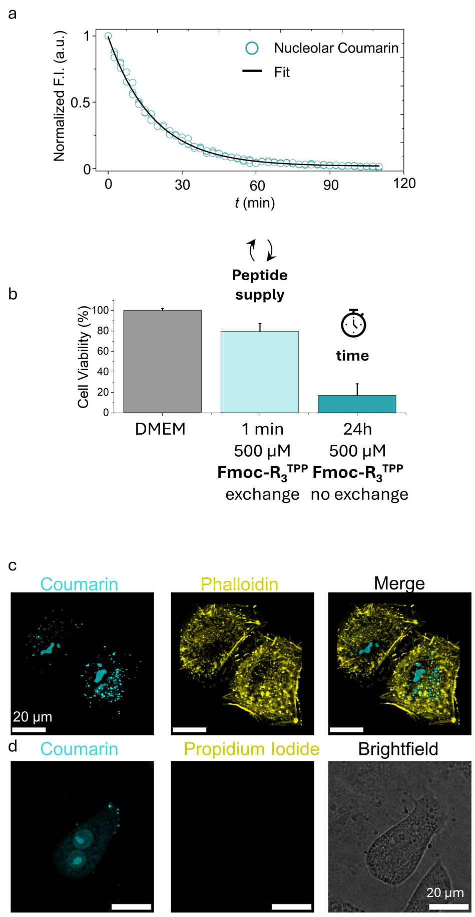

Figure S22: Non-equilibrium steady-state condition character of complex coacervates. **a**, Quantification of nucleolar Coumarin signal over time after medium exchange. The average of n=3 droplets from two cells are fitted and depicted

as black line. **b**, Cell viability assay of recovery experiment. Cells were either treated with DMEM, 500 μM of **Fmoc-R$_3^{TPP}$** for 1 min after which the media was exchanged to DMEM or for 24 hours with 500 μM of **Fmoc-R$_3^{TPP}$**. Error bars represent standard deviations from n=6 replicas. **c**, 3D representation of phalloidin (yellow) and Coumarin (cyan) of fixed and co-stained cells. **Fmoc-R$_3^{TPP}$/Coum-R$_3^{TPP}$** of 250 μM after 2 h of incubation. **d**, CLSM of 250 μM **Fmoc-R$_3^{TPP}$/Coum-R$_3^{TPP}$** treated A549 cells to elucidate cellular uptake mechanism in the presence of PI.

References

(1) Poprawa, S. M.; Stasi, M.; Kriebisch, B. A. K.; Wenisch, M.; Sastre, J.; Boekhoven, J. Active Droplets through Enzyme-Free, Dynamic Phosphorylation. *Nature Communications 2024 15:1* **2024**, *15* (1), 1–11. https://doi.org/10.1038/s41467-024-48571-z.

(2) ALETRAS, A.; BARLOS, K.; GATOS, D.; KOUTSOGIANNI, S.; MAMOS, P. Preparation of the Very Acid-Sensitive Fmoc-Lys(Mtt)-OH Application in the Synthesis of Side-Chain to Side-Chain Cyclic Peptides and Oligolysine Cores Suitable for the Solid-Phase Assembly of MAPs and TASPs. *Int. J. Pept. Protein Res.* **1995**, *45* (5), 488–496. https://doi.org/10.1111/J.1399-3011.1995.TB01065.X.
